# Heat-not-burn technology affects plasma testosterone levels and markers of inflammation, oxidative stress in the testes of rats

**DOI:** 10.3389/ftox.2024.1515850

**Published:** 2025-01-20

**Authors:** Silvia Granata, Camilla Morosini, Maria Chiara Valerii, Ivan Fagiolino, Stefano Sangiorgi, Severino Ghini, Enzo Spisni, Fabio Vivarelli, Lucy C. Fairclough, Moreno Paolini, Donatella Canistro

**Affiliations:** ^1^ Department of Pharmacy and Biotechnology, Alma Mater Studiorum, University of Bologna, Bologna, Italy; ^2^ Department of Biological, Geological and Environmental Sciences, Alma Mater Studiorum, University of Bologna, Bologna, Italy; ^3^ Gruppo CSA—S.p.A., Rimini, Italy; ^4^ School of Life Sciences, University of Nottingham, Nottingham, United Kingdom

**Keywords:** heat-not-burn, oxidative stress, inflammation, DNA damage, testis

## Abstract

**Introduction:**

Heating tobacco products (HTPs) are advanced electronic cigarette models. Classified by the FDA as a modified-risk tobacco product and can be used as part of efforts to quit smoking. Using heat-not-burn (HnB) technology, these devices heat tobacco avoiding complete combustion. Although the levels of toxicants in the mainstream are significantly lower than those observed in tobacco smoke, some recent studies have raised concerns about potential health risks associated with their use, particularly regarding their effects on male gonadal function, which remain largely unexplored.

**Methods:**

Adult male Sprague-Dawley rats were exposed, whole body, 5 days/week for 4 weeks to HnB mainstream.

**Results:**

The expression of the cell cycle regulators Bax/Bcl-2 ratio is not affected, along with no changes in p-38. On the other hand, an increase in oxidative stress markers, including those associated with DNA damage, was observed in exposed animals, along with the induction of NF-kB dependent pro-inflammatory mediators: TNF-α, IL-1β, IL-6 and COX-2. Furthermore, inactivation of key androgenic enzymes, such as 3β-hydroxysteroid dehydrogenase and 17β-hydroxysteroid dehydrogenase, together with decreased testosterone synthesis suggest a potential impairment of male gonadal function.

**Discussion:**

The results indicate that animals exposed to HnB smoke show higher levels of oxidative stress markers, including those associated with DNA damage, as well as higher levels of pro-inflammatory cytokines. The impairment of some androgenic key enzymes and those related to the activity of seminiferous epithelium, together with the decrease in testosterone levels, suggest an impairment of gonadal function through the alteration of some cellular pathways typically associated with tobacco consumption.

## 1 Introduction

In recent years, some health concerns have been raised relating to the utilization of electronic cigarettes, outlining the risks linked to the onset of respiratory and cardiovascular diseases and even cancer (Vivarelli et al., 2022; Vivarelli et al., 2024; Goebel et al., 2023; Tang et al., 2019; Lee et al., 2018; Fountoulakis et al., 2023). Heating tobacco products (HTPs) or heat-not-burn (HnB) devices feature technology that heats tobacco without combustion, with the stated purpose of significantly reducing the emission of toxic compounds while significantly reducing the health risks associated with tobacco consumption (Upadhyay et al., 2023). HnBs can be considered hybrids between conventional cigarettes (CCs) and electronic cigarettes (EC). While CCs volatilize nicotine through tobacco combustion at temperatures around 700°C, HnB heats ground tobacco that is reconstituted into sheets through contact with a metal blade as a heating element or a heating chamber where tobacco sticks are placed inside the device, and external resistive heaters made of stainless-steel tracks (Vivarelli et al., 2022). HTPs are presented to consumers as novel tobacco products or smokeless tobacco products, strengthening their cleanliness appeal, especially among younger people, where they are attracting growing interest (Simonavicius et al., 2019). Although the mainstream of HnB systems contains several classes of harmful chemicals typically recorded in traditional tobacco smoke, such as toxic aldehydes and carcinogenic polycyclic aromatic hydrocarbons (PAHs), concentrations were found to be significantly lower (Vivarelli et al., 2022; Vivarelli et al., 2024; Goebel et al., 2023; Tang et al., 2019; Lee et al., 2018; Fountoulakis et al., 2023; Upadhyay et al., 2023; Simonavicius et al., 2019; Auer R et al., 2017). For this reason, in the United States of America, they are regulated as non-combustible cigarettes and FDA authorized the marketing of HnB devices as “modified risk tobacco products” (FDA, 2024). However, data from previous studies are raising some toxicological concerns, especially related to the pro-oxidant and pro-inflammatory effect of HnB mainstream exposure (Vivarelli et al., 2022; Vivarelli et al., 2024; Goebel et al., 2023; Tang et al., 2019; Lee et al., 2018; Fountoulakis et al., 2023) and recent preclinical studies have shown that exposure to HnB tobacco smoke could be associated with some health concerns typically elicited by traditional smoking (Vivarelli et al., 2022; Fried and Gardner, 2020; Sawa et al., 2022; Koike et al., 2022; Gu et al., 2023). The high content of polyunsaturated fatty acids in membranes, the limited antioxidant capacity as well as the ability of spermatozoa to generate reactive oxygen species (ROS) make testis particularly susceptible to oxidative damage. ROS contribute to testicular inflammatory response by the activation of IL-1β mediated cascade affecting spermatogenesis and testosterone synthesis (Wang et al., 2023). In this regard, oxidative stress caused by CCs is a common feature underlying male gonadal impairment (Martin-Hidalgo et al., 2019). Since tobacco smoking still remains one of the leading causes of male infertility (Osadchuk et al., 2023), due to the presence of nicotine, tar, carbonic monoxide, PAHs and heavy metals (Dai et al., 2015; Duca et al., 2019; Bastianini et al., 2021), and as these are also partially present in HnB mainstream products, the present study aimed to investigate in a rat model the effects of HnB tobacco exposure on testicular function by focusing the expression of pro-inflammatory NF-κB target genes and oxidative stress markers, including those associated with DNA damage. The putative alterations of 3β- and 17β-hydroxysteroid dehydrogenase, two key enzymes of steroidogenesis typically affected by CCs exposure, were studied along with the plasmatic concertation of testosterone.

## 2 Materials and methods

### 2.1 Heat not burn (HnB) device

The Heat-not-Burn THS 2.2 model manufactured by PMI was used in the present study to deliver tobacco smoke. The device and the tobacco sticks (HEETS Bronze) used are commercially available.

### 2.2 Chemical analysis on HnB smoke

Chemical analysis on HnB smoke was performed as previously described (Vivarelli et al., 2022). The compounds analyzed in this study and the methods used are reported and listed in the Table 1.

**TABLE 1:** Analysis methods for chemical characterization.

Chemical classes	Methods
Polycyclic Aromatic Hydrocarbons (PAHs)	DM 05/05/2015 GU n128 05/06/2016
Volatile Organic Compounds (VOCs)	UNI EN ISO 16017–1:2002
Aldehydes	EPA 8315A 1996
Nicotine	NIOSH 2551 1998

### 2.3 Animal exposure

To ensure an appropriate O_2_/CO_2_ and O_2_/N_2_ ratio, we sampled the air in the exposition chamber by the use of a Hamilton airtight syringe (30 mL), which was immediately transferred into a 5 mL capped vial and injected onto a GC/MS (QP-2010 Plus, Shimadzu, Japan) system equipped with a RTX-WAX column (30 m, 0.25 mm i. d., 0.25 μm film thickness, Restek, United States of America). This was interfaced with a computerized system for data acquisition (Software GC–MS Solution V. 2.5, Shimadzu, Japan) (Cardenia et al., 2018; Vivarelli et al., 2021). 7-week-old Sprague Dawley male rats (ENVIGO RMS S. r.l., San Pietro al Natisone, Udine, Italy) were housed under standard conditions (12 h light-dark cycle, 22°C, 60% humidity). After 2 weeks of acclimatization, animals were randomly assigned to the control or exposed experimental unit. Animals enrolled to the study had no significant difference in weight. The animals exposed to HnB smoke were placed in a chamber (38 × 26.5 × 19 cm) with a capacity of 19 L and were exposed via the whole-body. Each chamber containing two animals was associated with a pump (0.18 kW; 1.4/1.6 A; 230 V; 50/60 Hz) installed on one side of the chamber, while the HnB smoke was puffed on the other, generating the airflow into the chamber. After 1 week of acclimatization, the animals were separated into two groups, a control (n = 6 rats) and an exposed (n = 6 rats) group. Before the exposure session start, the exposure chambers were properly prepared by adding bedding to ensure better animal comfort and absorption of waste. Each exposure cycle consisted of the following puff profile: 5 s on, 15 s off, 5 s on, with an airflow of 4 L/min. The puff profile and flow rate were determined in accordance with previous studies on e-cigarettes. The devices were turned off, and the animals were exposed to the vapor in the chamber for 20 min. After this period, the chamber was opened, the walls were dried, and a new cycle could start. This procedure was carried out for a maximum of 3 h, allowing the daily consumption of 8 tobacco sticks per exposure chamber. The concentration of nicotine recorded in HnB smoke was significantly lower than the LC_50_ for vaporized nicotine in the rat model (2.3 mg/L). Daily treatments were scheduled for five consecutive days followed by two rest days, for 4 weeks. During the exposure, several identical devices were used, and while some were in operation, those just used were cleaned using the cleaning kits provided by the manufacturer after each use (Ardati et al., 2024). The cleaning of the blade and the area adjacent to it was carried out until all debris was completely removed and the blade appeared entirely clean (by eye inspection). The blade was then dried, and any tissue residue was removed to avoid unwanted burn phenomena. At the end of each session, the blade was inspected, and if any imperfections or damage were noticed, a request for replacement was made. The Animal Welfare Committee monitored the animal throughout the entire experimental program. Permit number: 2683015.

### 2.4 Tissue collection and sub-cellular fractions

Animals were injected with 100 mg/kg of Zoletil 100 i. p. to obtain a deep sedation and, the sacrifice was performed through decapitation. All procedures were set according to the animal welfare committee of the University of Bologna. Following sacrifice, testis were removed and rapidly frozen into liquid nitrogen then stored at −80°C. Testis were homogenized in 50 mM NaCl, 1 mM EDTA, 1% Triton-X-, and 20-mM TRIS-HCl pH 7.4, by using a IKA Ultra-Turrax homogenizer. The homogenized fraction was centrifuged for 65 min at 105,000 × g, after which the cytosolic fraction (supernatant) was collected. The pellet was resuspended in 0.1 M K_2_P_2_O_7_, 1 mmol/L EDTA (pH 7.4) and centrifuged again for 65 min at 105,000 × g to give the final microsomal fraction. Full details are reported previously (Vivarelli et al., 2016). Protein extraction for immunoblotting analysis was performed by the use of T-PER. Tissue protein extraction reagent (Thermo Scientific, Waltham, MA, United States) was used following the manufacturer’s procedures. Halt Protease and Phosphate inhibitor cocktail (Thermo Scientific) was added in accordance with the datasheet recommendations. The blood collection for plasma assays were performed via venous access and samples collected in EDTA tubes and stored at 4°C prior to use. We set our collecting procedure between 9.00 and 10.00, taking care not to create time differences in the various groups that could have influenced the results and as a matter of practice, subjects are assigned to a blood collecting session randomly.

### 2.5 Protein concentration

Protein concentration from cytosolic and microsomal fractions was determined according to the method described by Lowry et al. (1951) using bovine serum albumin as a standard and diluting samples 200 times to provide a suitable protein concentration. Protein quantification of the tissue protein fraction was performed using a Pierce BCA protein assay kit (Invitrogen Thermo Scientific). The Pierce BCA (Bicinchoninic Acid) Protein Assay is a colorimetric method used to quantify the concentration of proteins in a sample. The assay is based on the reduction of Cu^2^⁺ to Cu⁺ by proteins in an alkaline environment, followed by the complexation of Cu⁺ with bicinchoninic acid to form a colorimetric complex that absorbs light at 562 nm. The intensity of the colour is proportional to the protein concentration. A standard curve was used to determine the protein concentration of the samples.

### 2.6 Measures of reactive oxygen species (ROS) by the 2′,7′-dichlorofluorescein diacetate (DCFH-DA) assay

2′,7′-dichlorofluorescein diacetate (DCFH-DA) was used as probe for the estimation of ROS content in tissue homogenate. An aliquot of the sample was incubated with DCFH-DA (100 μM) at 37°C for 30 min. The reaction was terminated by chilling the reaction mixture in ice. The formation of the oxidized fluorescent derivative (DCF) was monitored at excitation and emission wavelengths of 488 and 525 nm, respectively, using a fluorescence spectrophotometer. The DCF was quantified using a standard curve as previously reported (Rodrigues et al., 2005) with a few modifications (Cirillo et al., 2019) and was expressed as molar concentration of DCF per mg of protein.

### 2.7 Malondialdehyde (MDA) lipid peroxidation assay

200 μL of cytosol samples were mixed with 1 mL of acetic acid (20%) then 200 μL of 8% sodium dodecyl sulphate; the pH was adjusted to 4.0 using NaOH. Thiobarbituric acid (TBA) (40 mM, 1.5 mL) was added to samples, and they were placed in a boiling water bath for 60 min. After cooling, 3 mL of n-butanol was added, and samples were centrifuged at 10,000 × g for 15 min. The clear butanol fraction was isolated and the absorbance at 532 nm was measured. A standard curve was drawn from hydrolyzed 1,1,3,3-tetramethoxypropane (TEP) dissolved in water (Vivarelli et al., 2018).

### 2.8 Protein carbonyl groups (PC) assay

1 mL of cytosol samples were mixed with a 200 μL of 10 mM DNPH solution and were placed in the dark at room temperature (RT) for 60 min, with vortex-mixing every 15 min. A volume of 1.2 mL of 20% TCA solution was added and samples were chilled on ice for 15 min. Samples were centrifuged at 10,000 × g in a tabletop microcentrifuge for 5 min at 4°C and the protein pellet was washed with 1 mL of 20% TCA. Protein pellets were resuspended with 1 mL of 1:1 (v/v) ethanol:ethyl acetate and mixed by vortexing in order to remove any free DNPH. Protein pellet was resuspended in 1 mL of 6 M guanidine hydrochloride at 37°C for 30 min with vortex mixing. Carbonyl contents can be determined from the peak absorbance at 366 nm by using a molar absorption coefficient of 22,000 M^−1^ cm^−1^ (Vivarelli et al., 2019a).

### 2.9 8-Hydro-2-Deoxyguanosine (8-OHdG) assay

The test was performed following the manufacturer’s instructions (DNA/RNA oxidative damage ELISA Kit by Cayman Chemicals, Ann Harbor, MI, United States). As previously reported (Vivarelli et al., 2024), the DNA was extracted from tissue biopsies by the use of AllPrep DNA/RNA Kit (QIAGEN, Venlo, Netherlands; see also Section 2.8), following the datasheet recommendations. Nucleosides were obtained using the DNA Degradase Plus Kit purchased from Zymo Research Irvine, CA, United States. The Cayman DNA Damage ELISA Kit is a sensitive assay used to measure levels of DNA damage in samples, typically by detecting DNA lesions such as 8-hydroxy-2′-deoxyguanosine (8-OHdG), which is a biomarker of oxidative DNA damage. The assay is based on the use of a pre-coated ELISA plate. 50 µL of standards or samples to the appropriate wells of the plate. The standards are used for generating the calibration curve and for quantification. The provided plate was incubated at 37°C for 1 h. This allows the DNA damage (e.g., 8-OHdG) to bind to the antibody on the plate. Each well was then washed with a washing buffer solution provided by the company and 50 µL of streptavidin-HRP solution was added to each well and the plate incubated at room temperature for 30 min with gentle shaking on a plate shaker. The reaction was blocked using a stop solution and the absorbance was recorded at 450 nm using a TEACAN GENios plate reader within 10 min after adding the stop solution. The amount of DNA damage (e.g., 8-OHdG) is directly proportional to the colour intensity, and absorbance readings can be used to determine the concentration of the biomarker.

### 2.10 Antioxidant enzymes activity

Several enzyme activities were determined within cytosolic fraction as previously described (Granata et al., 2023). Details on the procedures are briefly described below.

### 2.11 Catalase (CAT)

30 mmol of H_2_O_2_ was added to the reaction mixture, constituted by 50 mM potassium phosphate buffer and cytosol. The decomposition of the substrate was measured at 240 nm and catalase activity was expressed as mol of H_2_O_2_ consumed per minute per mg protein using a molar extinction coefficient of 43.6 mM^−1^ cm^−1^. Activity is reported in μmol mg^−1^min^−1^.

### 2.12 NAD(P)H:quinone reductase (NQO1)

NQO1 activity was assayed spectrophotometrically at 600 nm by monitoring the reduction of the blue redox dye DCPIP to its colorless form, using NADPH as the hydrogen donor. The assay mixture contained 50 mm-Tris–HCI (pH 7.5), 1 mm-NADPH and 40 μm-DCPIP. Enzymatic activity was calculated using the extinction coefficient of DCPIP (22.1 per mM^−1^ cm^−1^), and expressed as nmol DCPIP reduced/min per mg protein.

### 2.13 Superoxide dismutase (SOD)

The specific activity was assayed spectrophotometrically at 320 nm by monitoring the generation of adrenochrome, one of the main products of epinephrine autoxidation at pH 10.2. Briefly, the activity was assayed spectrophotometrically at 320 nm by monitoring the formation of adenochrome (ε = 4.02 per mM^-1^ cm^−1^). Activity is expressed in nmol/(mg × min).

### 2.14 Oxidized glutathione reductase activity (GSSG-red)

GSSG-red was measured by adding 1.5 mM NADPH into an assay cuvette containing 50 mM potassium-phosphate buffer, 1 mM EDTA, cytosol sample, and 20 mM GSSG. The generation of NADP^+^ from NADPH, during the reduction of GSSG, was recorded at 340 nm for 5 min at 37°C. The GSSG reductase was calculated with the extinction coefficient of 6.22 mM^−1^ cm^−1^ and expressed as μmol of NADPH consumed per minute per milligram of protein from cytosol (μmol mg^−1^ min^−1^).

### 2.15 Glutathione peroxidase (GSH-Px)

The reaction mixture containing 50 mM potassium phosphate, 1 mM EDTA, cytosol sample, 10 mM GSH, GSSG reductase (2.4 U/mL) and 1.5 mM NADPH was incubated at 37°C for 5 min, followed by the addition of tert-butylhydroperoxide. The NADPH consumption was followed at 340 nm. GSH peroxidase was calculated by using an extinction coefficient of 6.22 mM^−1^ cm^−1^ and expressed as nmol of NADPH consumed per minute per milligram of protein from cytosol.

### 2.16 Xanthine oxidase (XO)

The XO activity was measured spectrophotometrically by the formation of uric acid by the xanthine through the increase of absorbance recorded at 290 nm. The assay mixture containing 50 mM sodium phosphate buffer (pH 7.8) and hypoxanthine 50 μM and the cytosol fraction (50 uL) was incubated at 37°C for 5 min. The reaction was stopped by the addition of 0.1 mL of TCA and the mixture was centrifuged at 10,000 g for 10 min. The absorption at 290 nm was carried out using the clear supernatant fraction obtained. Blank was run in parallel with the same mixture without xanthine. The activity was calculated on the basis of the molar extinction coefficient for uric acid (7.6 mM^−1^ cm^−1^).

### 2.17 Testicular androgenic enzymes (3β-HSD; 17β-HSD)

3β-hydroxysteroid dehydrogenase (3β-HSD) and 17β-hydroxysteroid dehydrogenase (17β-HSD) in cytosolic fraction were assessed photometrically following the reduction of NAD to NADH at 399 nm at 25°C, pH 8.9. Dehydroepiandrosterone (DHEA) or testosterone were used as substrates for 3β-HSD or 17β-HSD, respectively. 3β-HSD activity was measured mixing 200 μL of cytosol with 100 mM sodium pyrophosphate buffer (pH 8.9) and 0, 01% of dehydroepiendrosterone ethanol solution. The enzyme activity was measured after addition of 6 mM of NAD to the tissue supernatant mixture in a cuvette at 340 nm against a blank (without NAD). One unit of enzyme activity is the amount causing a change in absorbance of 0.001/min at 340 nm (Omar et al., 2018).

### 2.18 Sorbitol dehydrogenase (SDH) and lactate dehydrogenase (LDH)

The LDH assay is based on the inter-conversion of lactate and pyruvate and the SDH assay is based on inter-conversion of Fructose and Sorbitol. During the reduction of pyruvate or fructose, an equimolar amount of NADH is oxidized to NAD. The oxidation of NADH results in a decrease in absorbance at 340 nm. The rate of decrease of absorbance at 340 nm is directly proportional to LDH or SDH activity in the sample, where one unit of LDH or SDH activity is defined as 0.1 μmol of substrate transformed/minute at pH 7.6 at 25°C.The SDH enzymatic activity was determined as the amount of fructose reacted per unit of time, and it was measured by recording the absorbance decrease due to NADH oxidation. The reaction was carried out in 1 mL final volume, which contained 0.75 mL of Tris buffer (111 mM, pH 7.5), 0.05 mL of NADH (1.8 mM) and 0.1 mL of cytosol. The solutions were mixed and incubated at 25°C for about 5 min. The reaction was started by adding 0.1 mL of 66.6 mM fructose solution (Vivarelli et al., 2019b). Cytosolic LDH was based on the interconversion of lactate and pyruvate. During pyruvate reduction, an equimolar rate of NADH is oxidized to NAD resulting in an absorbance decrement at 340 nm (Aly and Khafagy, 2014). The reaction was carried out in 1 mL final volume containing 200 mM tris buffer, 30 mM tris buffer pyruvate solution, 6.6 mM NADH and the reaction start with 0.2 mL of cytosol. The oxidation of NADH was recorded spectrophotometrically at 340 nm (molar extinction coefficient 6.22 M^−1^ cm^−1^). The final enzyme activity was normalized considering the protein concentration of cytosol samples loaded and expressed as μmol min^−1^ mg of protein^−1^.

### 2.19 Plasma testosterone levels

Whole blood samples were collected from the tail vein within 2 min of handling. Blood samples were collected in EDTA tubes, stored at 4°C and centrifuged at 1,000 × g within 30 min of collection. Testosterone levels were determined using a kit based on a competitive-ELISA detection method purchased by Antibodies (Cambridge, United Kingdom). 50 μL of samples or standard were incubated with 50 μL of Biotin-labeled Antibody at 37°C for 45 min. The plate was aspirated and washed carefully and then 100 μL of HRP-streptavidin conjugate solution was added in each well and incubated for 30 min at 37°C. The plate was washed carefully and 90 μL of 3,3′,5,5′-tetramethylbenzidine (TMB) chromogenic solution was added and the plate was incubate for 20 min at 37°C. 50 μL of stop solution was added and the O.D. was recorded at 450 nm using a TECAN GENios plate reader. During the reaction, the target in the samples (or standard) competes with a fixed amount of the target on the solid phase supporter for sites on the biotinylated detection antibody specific to the target. The concentration in the sample is determined by comparing the OD of the samples to the standard curve.

### 2.20 Western blot analysis

The following procedure was used to study the change of protein expression in testis tissue. Testis protein extraction was performed using T-PER Tissue protein extraction buffer (Thermo Scientific, Waltham, MA, United States) and Halt Protease and Phosphate inhibitor cocktail (Thermo Scientific) following the manufacturer’s recommendation. Proteins were separated in one dimension on Bolt 4%–12% Bis-tris Plus gels (Invitrogen Thermo Scientific) using a mini protean vertical gel electrophoresis mini-tank module (Invitrogen Thermo Scientific). Electrophoresis was performed for 3 h at 150 V at room temperature using the MOPS (1 M Tris, 2% SDS, 20 mM EDTA; pH 7.7) or MES (1 M Tris, 20 mM EDTA and 2% SDS; pH 7.3) purchased by Life Technologies, Thermo Scientific. A mixture of pre-stained protein standards (SeeBlue Plus-2 by Life Technologies, Thermo Scientific) with molecular weights ranging from 198 to 3 kDa, was loaded as molecular weight marker. Proteins were transferred onto a 0.2 μm pore size nitrocellulose membrane (Novex-Life Technologies, Thermo Scientific) at 10 V for 2 h using 25 mM Tris, 190 mM glycine 20% methanol as transfer buffer. Non-specific binding sites on the membrane were blocked with Pierce Clear Milk Blocking buffer (Thermo Scientific). Primary antibodies were diluted in TBST skimmed milk 5% buffer and the incubation was performed overnight at 4°C using an orbital shaker followed by 2 h at room temperature with the secondary horseradish peroxidase (HRP)-linked antibody (Goat Anti-Mouse or Anti-Rabbit IgG Peroxidase Conjugated by Thermo Scientific). Proteins that bound the antibody were visualized by chemiluminescence procedures (Clarity Western ECL substrate Bio-Rad). Immunocomplexes were visualized via chemiluminescence using a Chemidoc MP Imaging System (Bio-Rad Laboratories, Hercules, CA, United States) and analyzed with the software ImageJ 1.7.0. The gels were each run twice, and the results represent the average from two different Western blots. The signal intensity of each lane was normalized to the α-tubulin loading control. Each experiment was performed at least twice, and presented data are means ± SD of relative intensity units. The primary antibody working dilutions were determined following the manufacturer’s recommendations: mouse monoclonal antibody for mouse α-tubulin (1:1,000; IgG isotype for WB application) (Thermo Scientific cat n. 14–4,502–82), Rabbit NRF2 (1:500; IgG isotype for WB application) (ABClonal ct n A0674); rabbit polyclonal antibody to IL-6 (1:500; IgG isotype for WB application) (ABClonal ct n A0286); rabbit polyclonal antibody to IL-8 (1:500; IgG isotype for WB application) (ABClonal ct n A02541); rabbit polyclonal antibody to NF-κB (phospho-Ser 536) (1:500; IgG isotype for WB application) (ABClonal ct n AP1294), rabbit polyclonal antibody to Nf-kB (1:500; IgG isotype for WB application) (ABClonal ct n A2547); rabbit polyclonal antibody to TNF-α (1:500; IgG isotype for WB application) (ABClonal ct n A2547); rabbit polyclonal antibody to IL-1β (1:500; IgG isotype for WB application) (ABClonal; ct n A17376); rabbit polyclonal antibody to COX-2 (1:500) (Bioss ct n BS0732); rabbit polyclonal antibody to H2AX (phospho Ser 139) (ABClonal ct n AP009) (1:500); rabbit polyclonal antibody to H2AX (1:500 IgG isotype for WB application) (ABClonal: ct n A11361); rabbit polyclonal antibody to OGG-1 (1:500 IgG isotype for WB application) (Elabscience ct n E-AB-60300); rabbit polyclonal antibody to XPC (Cloud-Clone Corp PAA473rA01) (1:500 IgG isotype for WB application); rabbit polyclonal antibody to CYP 1A1 (1:500 IgG isotype for WB application) (Cloud-Clone Corp ct n PAD29Ra01); rabbit polyclonal antibody to SIRT-1 (1:500 IgG isotype for WB application) (ABclonal; A11267) rabbit polyclonal antibody to SOD-1 (1:500) (Choesion Bioscience; CPA9446); rabbit polyclonal antibody to CAT (1:500) (Cloud-Clone Corp; PAC418Ra01).

### 2.21 Cytochrome P450 (CYP)-linked monooxygenases

Pentoxyresorufin O-dealkylase (CYP2B1/2), ethoxyresorufin O-deethylase (CYP1A1) and methoxyresorufin O-demethylase (CYP1A2) were estimated using pentoxyresorufin, ethoxyresorufin and methoxyresorufin as substrates, respectively, and monitoring the formation of resorufin as final reaction product was monitored by the use of Perkin Elmer LS 50 B Luminescence spectrometer. The reaction mixture consisted of 0.025 mM MgCl_2_, 200 mM pentoxyresorufin, 0.32 mg of liver microsomal proteins, and 130 mM NADPH in 2.0 mL of 0.05 M Tris-HCl buffer (pH 7.4). Resorufin formation at 37°C was calculated by comparing the rate of increase in relative fluorescence to the fluorescence of known amounts of resorufin (excitation 562 mm, emission 586 nm). Ethoxyresorufin O-deethylase and methoxyresorufin O-demethylase activities were measured in the same way as described for the pentoxyresorufin assay, except that the concentration of substrates was 1.7 mM ethoxyresorufin and 5 mM methoxyresorufin assays were carried out using the microsomal fraction of the tissue (Budriesi et al., 2018).

### 2.22 Statistical analysis

Data were evaluated by Shapiro–Wilk tests to confirm normality of distribution and by Grubb’s test to identify outliers. Statistical analysis was performed using the two-tailed unpaired *t*-test or Mann-Whitney test in case of non-normality distribution. All statistical analyses were performed using GraphPad 9 software (San Diego, CA, United States). Results are expressed as means ± SD. Experiments were performed on n = 6 tissue samples from control group and n = 6 tissue samples from HnB group. Immunoblotting analyses were performed on n = 4 tissue samples from control group and n = 5 tissue samples from HnB group. p values < 0.05 were considered statistically significant: *p < 0.05; **p < 0.01; ***p < 0.001. Data are presented as mean means ± SD.

## 3 Results

### 3.1 Chemical characterization of HnB mainstream

HnB mainstream was characterized using GC-MS, which revealed the presence of aldehydes (e.g., formaldehyde, acetaldehyde) and polycyclic aromatic hydrocarbons (PAHs) (Table 2). These compounds are indicative of thermal degradation and tobacco pyrolysis.

**TABLE 2:** Chemical characterization of HnB mainstream smoke.

Aldehydes	μg/stick ±S.D.	PAHs	μg/stick ±S.D.	VOCs and nicotine	μg/stick ±S.D.
Formaldehyde	3.00 ± 0.45	Naphthalene	<0.1	VOCs	1.09 ± 0.27
Acetaldehyde	14.0 ± 2.1	Acenaphthylene	<0.1	Benzene	0.078 ± 0.020
Propionaldehyde	1.00 ± 0.15	Acenaphthene	<0.1	Ethylbenzene	0.078 ± 0.020
Butyraldehyde	1.00 ± 0.15	Fluorene	<0.1	Toluene	0.183 ± 0.046
Crotonaldehyde	<1	Phenanthrene	2.4 ± 1.0	Xylenes	0.56 ± 0.14
Acrolein	<1	Anthracene	<0.1	Alkylbenzenes	0.189 ± 0.047
		Fluoranthrene	6.2 ± 2.7	Nicotine	113 ± 26
		Pyrene	4.4 ± 1.9		
		Benzo(a)anthracene	1.7 ± 0.7		
		Cyclopenta (c,d)pyrene	0.4 ± 0.2		
		Chrysene	1.8 ± 0.8		
		Benzo(b)+(j)fluoranthene	1.1 ± 0.5		
		Benzo(k)fluoranthene	0.4 ± 0.2		
		Benzo(e)pyrene	0.7 ± 0.3		
		Benzo(a)pyrene	0.4 ± 0.2		
		Indeno (1,2,3,c,d)pyrene	0.3 ± 0.1		
		Dibenzo (ac)+(ah)anthracene	<0.1		
		Benzo (g,h,i)perylene	0.5 ± 0.2		
		Dibenzo (a,l) pyrene	<0.1		
		Dibenzo (a,e)fluoranthene	<0.1		
		Dibenzo (a,e) pyrene	<0.1		
		Dibenzo (a,i) pyrene	<0.1		
		Dibenzo (a,h) pyrene	<0.1		

The data shows the chemical profile of the mainstream released by HnB. Toxic aldehydes and PAHs were detected as pyrolysis marker. Data are expressed as means ± SD of at least two replicates from two independent experiments.

### 3.2 Radical oxygen species (ROS) production and alternations of oxidative stress markers

**FIGURE 1:**
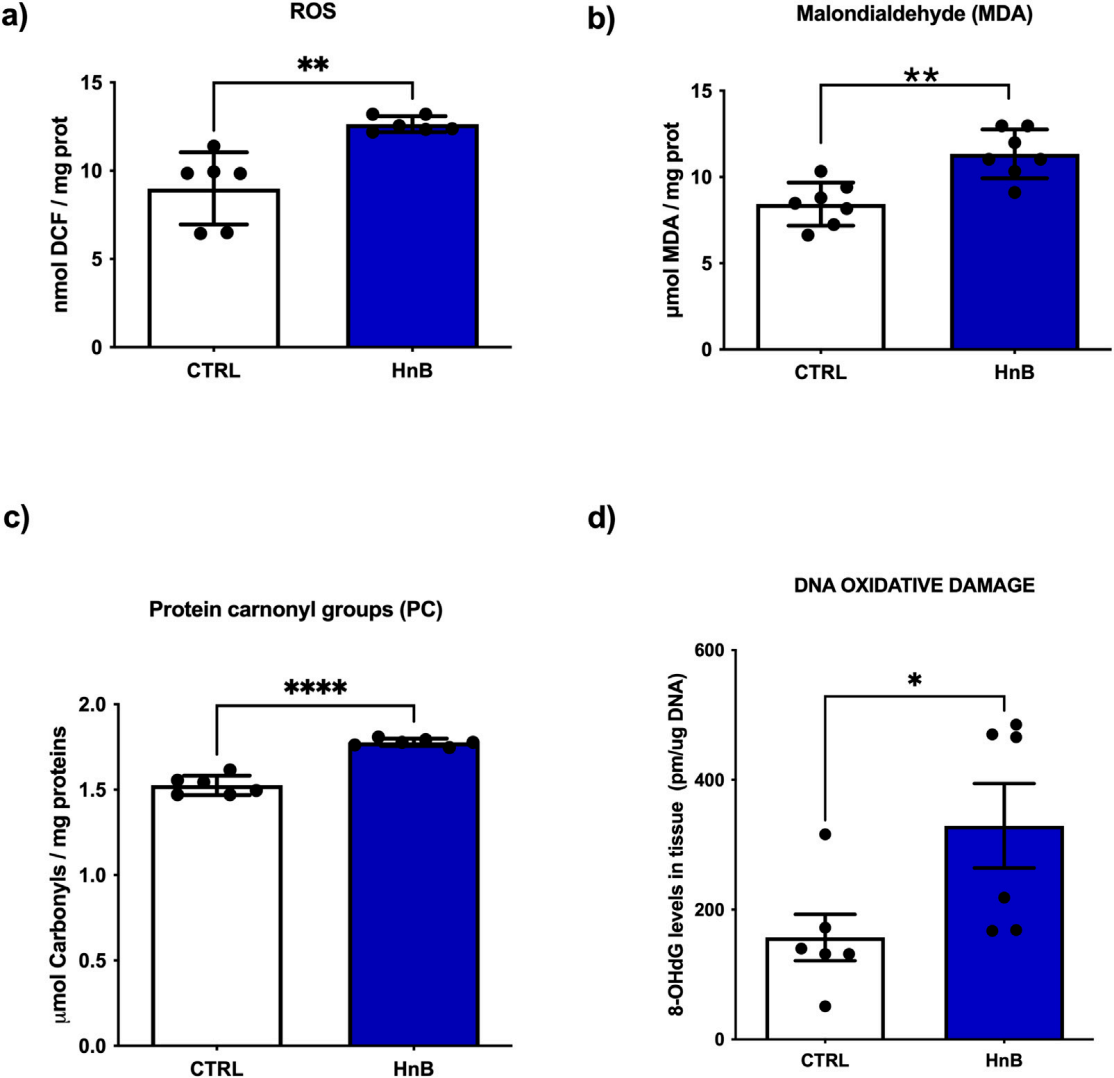
HnB smoke exposure increases oxidative stress markers in rat testis. The content of radical species, here measured with the DCFH-DA assay, is significantly higher in samples from exposed animals compared to controls **(A)**. HnB group shows an increase in MDA **(B)** and carbonyls **(C)** as markers of lipid and protein oxidation, respectively. Higher levels of 8-OHdG **(D)** as DNA damage marker are here reported in the exposed group compared to controls. Graphs report the means ± SD; *p < 0.05, **p < 0.01, *****p < 0.0001 two-tailed *t*-test.

A significant increase of ROS content was observed in the treated group compared to the control one (p < 0.01, Figure 1A). In addition, increased levels of malondialdehyde (MDA) (p < 0.01, Figure 1B) and protein carbonyl groups (PC) (p < 0.0001, Figure 1C), along with the higher levels of 8-OHdG (p < 0.05, Figure 1D) were also detected in the treated group.

### 3.3 DNA oxidative damage and activation of DNA repair enzymes

**FIGURE 2:**
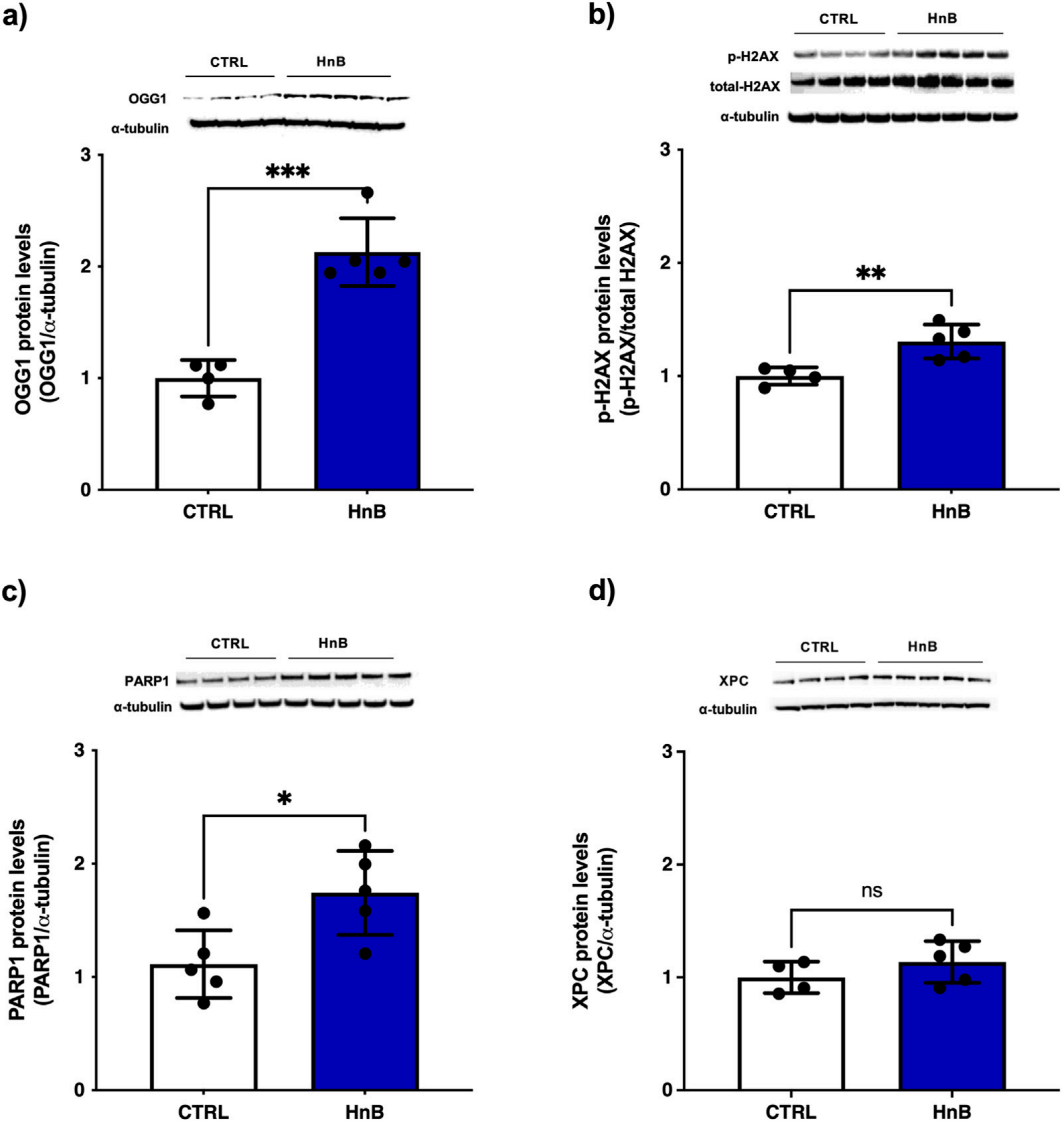
Animals exposed to HnB smoke show activation of the DNA repair machinery through induction of OGG-1 H2AX and PARP-1 in testes. The base excision repair system OGG-1 (∼47 kDa) **(A)**, ubiquitinated-H2AX (∼25 kDa) **(B)** and PARP-1 (∼89 kDa) **(C)** are significantly upregulated in HnB group compared to controls. No significant changes are observed in the expression of xeroderma pigmentosum group C (XPC) (∼26 kDa) **(D)**. The images are representative of two independent experiments. Bars report the means ± SD; *p < 0.05, **p < 0.01, ***p < 0.001, Welch’s two-sample two-tailed *t*-test.

Student’s t-test showed significantly higher levels of the DNA repair enzymes 8-oxoguanine DNA glycosylase-1 (OGG-1) (p < 0.001, Figure 2A), of the phosphorylation of histone H2AX at the Ser139 residue (p < 0.01, Figure 2B) and of poly (ADP-ribose) polymerase (PARP) (p < 0.05, Figure 2C) in the testis of rats exposed to HnB technology. Conversely, no significant changes of xeroderma pigmentosum group C protein complex (XPC) was observed (Figure 2D) in the exposed group of animals compared to the control one.

### 3.4 Activation of NRF2-mediated antioxidant response

**FIGURE 3:**
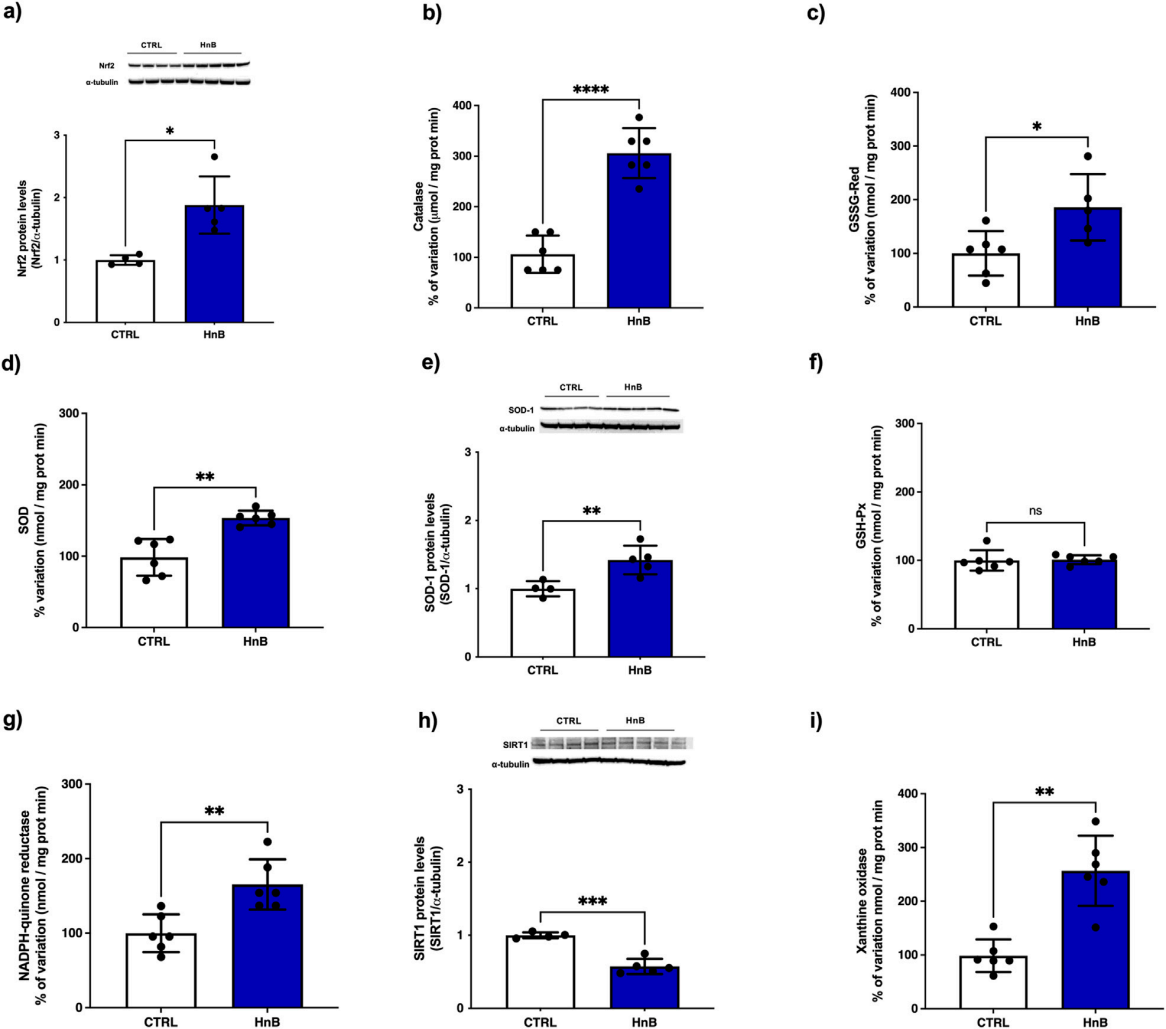
HnB smoke exposure induces the NRF2-mediated antioxidant response in rat testis. Samples from animals exposed to HnB show an increase of NRF2 (∼65 kDa) **(A)**, with an upregulation of CAT **(B)**, GSSG-Red **(C)**, SOD **(D)** and NQO1 **(G)** enzymatic activities. Immunoblot analysis confirm upregulation of SOD-1 (∼19 kDa) expression **(E)**. GSH-Px **(F)** showed no significant changes in enzymatic activity. SIRT-1 **(H)** expression (∼82 kDa) is downregulated. XO **(I)** expression as marker of OS shows a significant marked upregulation. The images are representative of two independent experiments. Bars represent the means ± SD; *p < 0.05, **p < 0.01, ***p < 0.001 ****p < 0.0001, Welch’s two-sample two-tailed *t*-test.

Increased levels of the transcription factor NRF2 (p < 0.05, Figure 3A), along with an upregulation of catalase (CAT) (p < 0.0001, Figure 3B), glutathione reductase (GSSG-Red), (p < 0.05, Figure 3C), superoxide dismutase (SOD) (p < 0.01, Figure 3D) and NADPH quinone reductase (NQO1) (p < 0.01, Figure 3G) enzymatic activities were detected in the exposed group. Moreover, higher protein levels of SOD-1 were also observed (Figure 3E). No significant changes in enzymatic activity of glutathione peroxidase (GSH-Px) (Figure 3F) were observed in testicular tissue. Interestingly, a downregulation of the protein levels of the sirtuin-1 (SIRT-1) (p < 0.001, Figure 3H) was observed after exposure to HnB mainstream. Moreover, an increased activity of the xanthine oxidase (XO) (p < 0.01, Figure 3I) was detected in the exposed animals compared to control animals.

### 3.5 Alterations of pro-inflammatory markers

**FIGURE 4:**
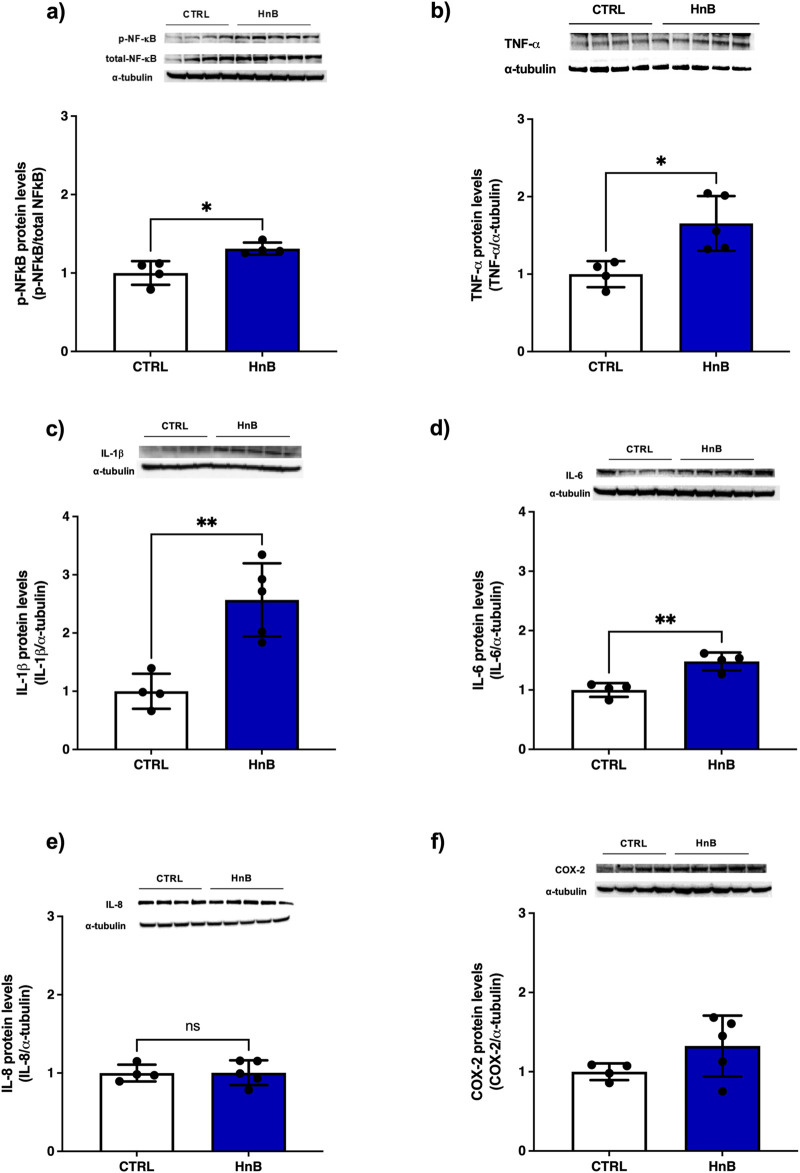
HnB smoking activates NF-κB mediated inflammatory response in rat testis. Exposed animals reported increased phosphorylation of NF-κB (p65 Ser 536) (∼61 kDa) **(A)**, along with the upregulation of TNF-α (∼25 kDa) cleaved form (∼17 kDa) **(B)**, IL-1β (∼17 kDa) **(C)**, IL-6 (∼26 kDa) **(D)**. Here, no significant changes in IL-8 expression (∼11 kDa) were observed **(E)**. Inducible COX-2 (∼50 kDa) **(F)** was significantly higher in HnB group compared to controls. The images are representative of two independent experiments. Bars represent the means ± SD; *p < 0.05, **p < 0.01, Welch’s two-sample two-tailed *t*-test.

Significantly higher levels of phosphorylated NF-κB protein levels (p < 0.05, Figure 4A) was observed in the testicular tissue of rats exposed to HnB mainstream compared to the control group. Furthermore, a significant increase in the expression of pro-inflammatory mediators TNF-α (p < 0.05, Figure 4B), IL-1β (p < 0.01, Figure 4C) and IL-6 (p < 0.01, Figure 4D) were also detected. No significant changes in the protein levels of IL-8 (Figure 4E) and COX-2 (Figure 4F) were observed.

### 3.6 Alterations of testicular physiology: 3βHSD and 17β-HSD activity as markers of steroidogenesis

**FIGURE 5:**
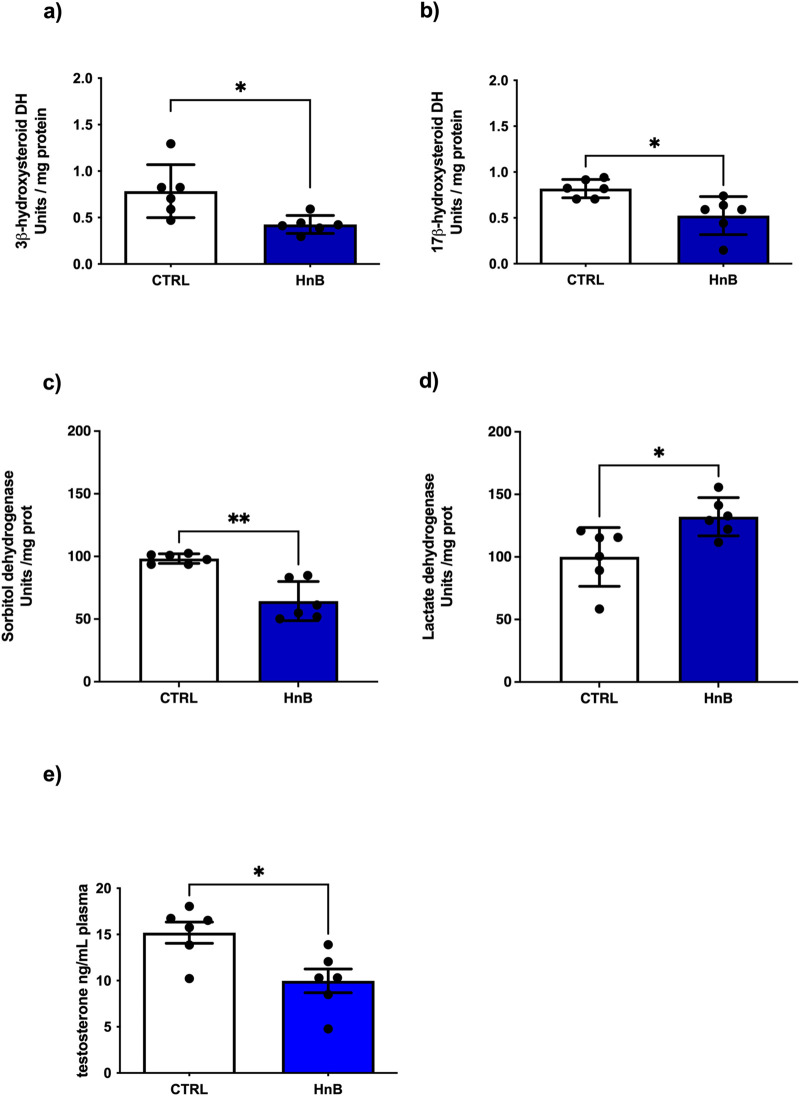
Effects of HnB smoke exposure on steroidogenesis and key functional enzymes of the testis. Data show an impairment of 3β-HSD **(A)** and 17β-HSD **(B)** as key enzymes involved in the testosterone synthesis. SDH **(C)** is downregulated while LDH **(D)** enzyme activity is significantly higher in HnB group samples compared to controls. Plasma testosterone levels measured here by ELISA are lower in exposed animals than controls **(E)**. The images are representative of two independent experiments. Bars represent the means ± SD; *p < 0.05, ***p < 0.001, Welch’s two-sample two-tailed *t*-test.

Since the 3β- and 17β-HSDs are key enzymes in the biosynthetic pathway of testosterone, a putative impairment in the activity of these enzymes may result in reduced steroidogenesis. A down-regulation of testicular 3β-HSD (p < 0.05, Figure 5A) and 17β-HSD (p < 0.05, Figure 5B), key enzymes involved in steroidogenesis, was observed in the rats exposed to HnB technology in comparison to the control group. These data are consistent with the significant decrease of plasma testosterone levels (p < 0.05, Figure 5E) in the treated group compared to the control group. Furthermore, a decrease in sorbitol dehydrogenase (SDH) (p < 0.001, Figure 5C), together with an increase in lactate dehydrogenase (LDH) activity (p < 0.05, Figure 5D), was also detected.

### 3.7 Protein alterations of cell cycle mediators

**FIGURE 6:**
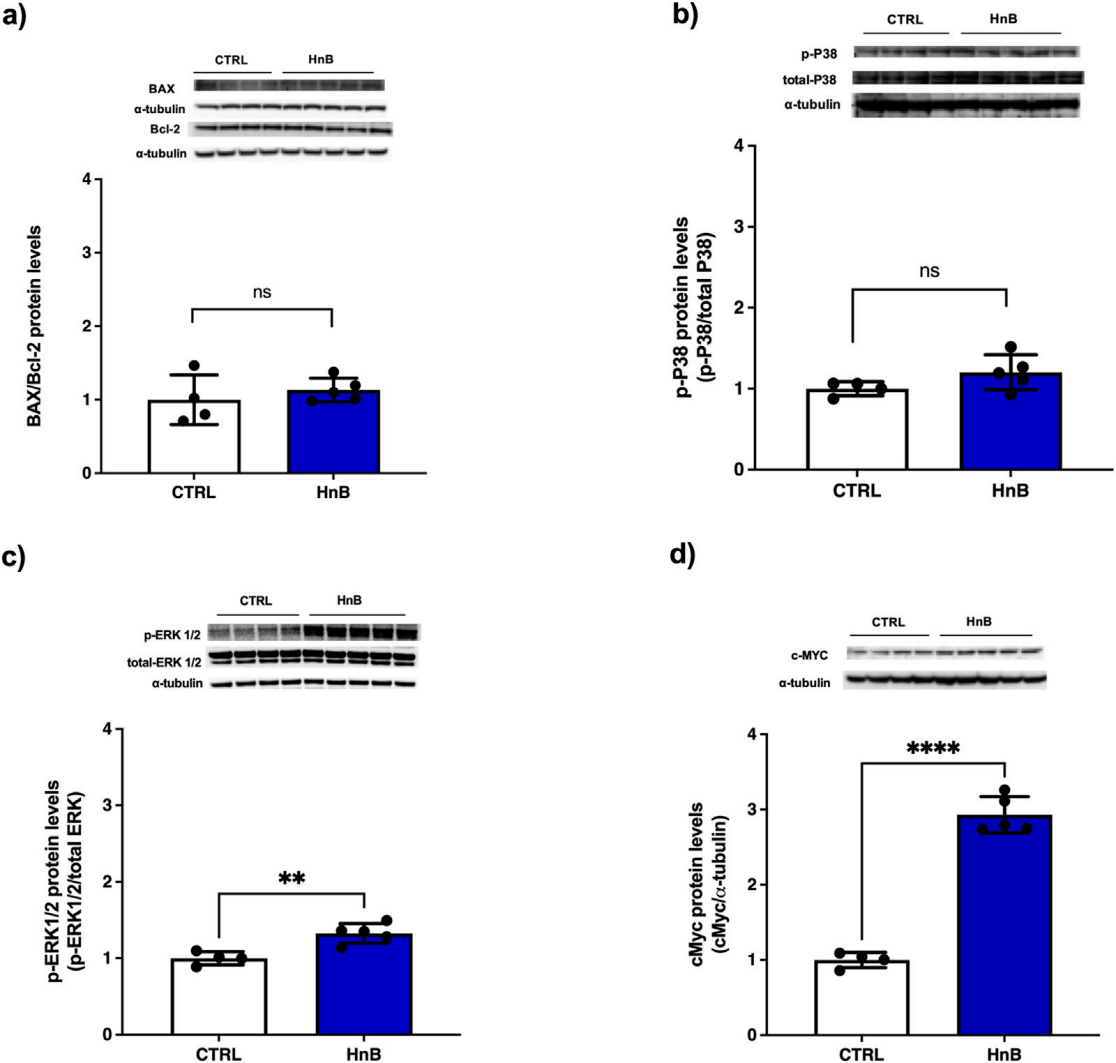
No significant changes were recorded for the Bax (∼21 kDa)/Bcl-2 (∼26 kDa) ratio **(A)** and for p38 (∼40 kDa) **(B)**. ERK1/2 (44–42∼kDa) was significantly activated by the treatment **(C)** and a marked up-regulation of c-MYC expression (∼49 kDa) was obtained in exposed animals **(D)**. The images are representative of two independent experiments. Bars represent the means ± SD; **p < 0.01; ****p < 0.0001, two-tailed *t*-test.

Nicotine induces apoptosis of Leydig cells, and this might be one of the important mechanisms behind nicotine-related urogenital disorders in men. Here we investigated the changes of Bax/Bcl-2 ratio and cMyc expression as a cell cycle regulator marker (Kim et al., 2005). Furthermore, the increment of ROS content along with the oxidative stress markers increase are suggestive of a putative activation of the p38, ERK MAPK signaling pathway (Torres, 2003)*.* No significant changes of BAX/Bcl-2 ratio (Figure 6A) and p-38 protein levels (Figure 6B) were observed. Conversely, higher levels of the phosphorylation of ERK (p < 0.01, Figure 3C) and a marked upregulation of c-MYC (p < 0.0001, Figure 3D) was detected in the testis of rats exposed to HnB mainstream compared to the control animals.

### 3.8 Alterations of cytochromes P450 (CYP) isoforms

**FIGURE 7:**
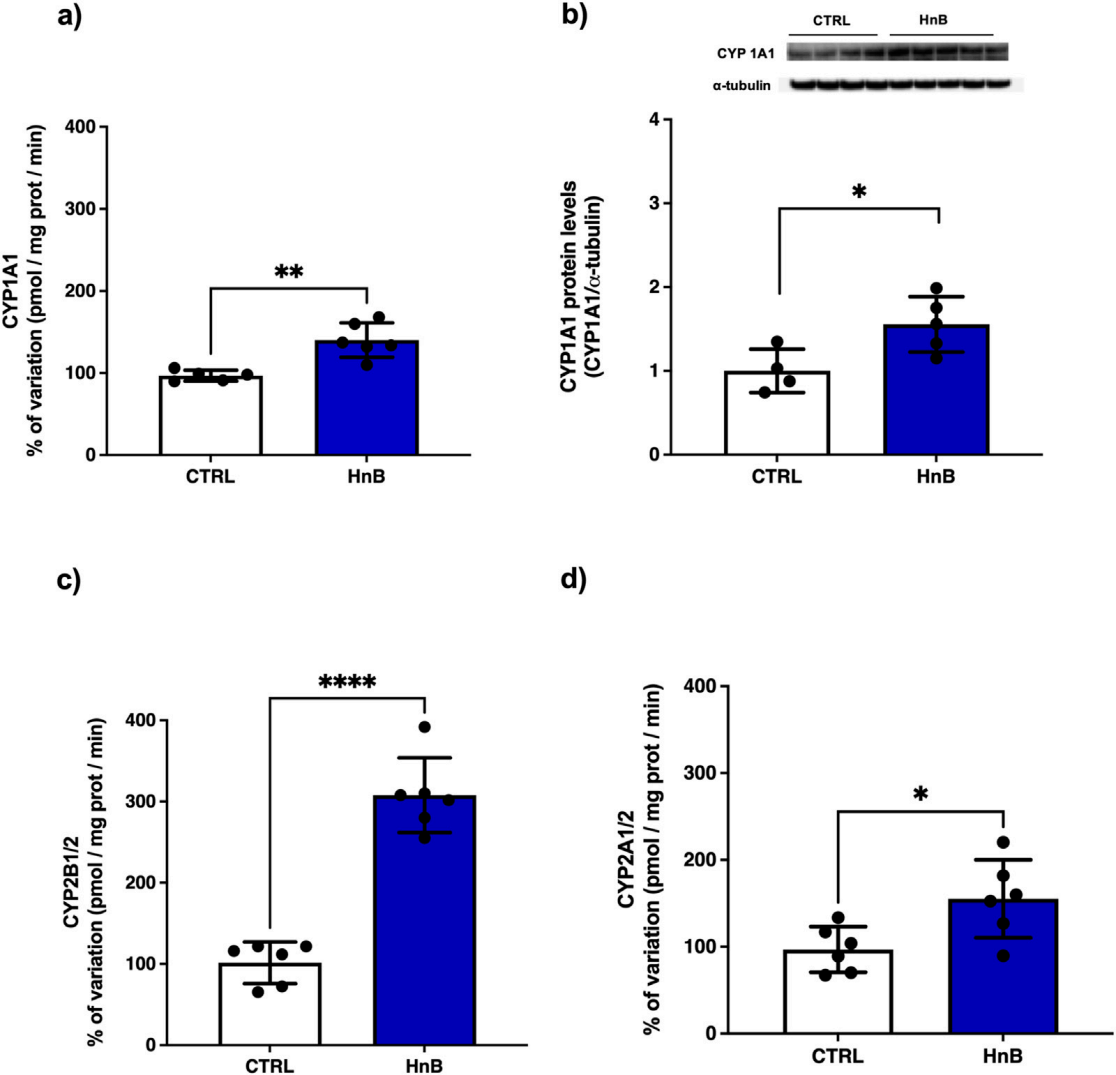
HnB smoke exposure boosts cytochrome-linked monooxygenase (CYPs) involved in PAH metabolism in rat testis. Data show an induction of CYP1A-dependent monooxygenases, activating aromatic amines, dioxins and PAHs **(A)** and the relative increase of protein expression (∼63 kDa) **(B)**. CYP2B1/2 (∼66 kDa) (also activating bupropion smoking-cessation drug) **(C)**; CYP2A 1/2 (∼50 kDa) (activating alcohol, nitrosamines, benzene, acetone, acrylamide) **(D)**. The images are representative of two independent experiments. Bars represent the means ± SD; *p < 0.05, ***p < 0.001, two-tailed *t*-test.

The presence of PAHs in HnB mainstream can change the expression of the main CYPs involved in the metabolism of these species (Pavek and Dvorak, 2008). The data analysis revealed an increased activity of CYP1A1 isoform (p < 0.05, Figure 7A), together with higher levels of CYP1A1 protein (p < 0.05, Figure 7B), in the treated group in comparison to the control animals. Similarly, a significant upregulation of CYP2B1/2 (p < 0.001, Figure 7C) and CYP2A1/2 (p < 0.05, Figure 7D) activities was also observed in testis of rats exposed to HnB mainstream.

## 4 Discussion

The deleterious effects of smoking on male gonadal function and fertility are well known (Sansone et al., 2018). Tobacco consumption leads to exposure to over 100 carcinogens and mutagens, including PAHs, aldehydes and heavy metals, which ultimately lead to DNA damage and testicular cytotoxicity (Sansone et al., 2018; Smith and Hansch, 2000). To date, very little is known about the toxicological effects of electronic cigarettes and HnB devices on testicles (Vivarelli et al., 2022). Our results, based on GC-MS analysis, indicate the presence of toxic by-products of tobacco thermal degradation in HnB mainstream, but at significantly lower concentrations than those recorded in conventional tobacco smoke.

PAHs are known to induce severe redox stress in cells and tissues, leading to the oxidation of nucleic acids, proteins, and lipids (Ju et al., 2022). Here we show a significant increase of reactive oxygen species (ROS) yield in animal testes exposed to HnB smoke. In addition, we observe higher levels of malondialdehyde (MDA), protein carbonyls and 8-hydroguanosine (8-OHdG) DNA adduct, as hallmarks of cellular lipid, protein and DNA oxidative damage, respectively. These results are of particular interest considering that DNA damage, largely due to oxidative stress, is associate with both male and female subfertility and is a major cause of defective sperm function (Bisht et al., 2017; Fabbri et al., 2015).

As expected, testis samples from exposed animals showed activation of DNA repair machinery, including the 8-oxoguanine glycosylase (OGG1), a base excision repair enzyme responsible for the recognition and removal of 8-OHdG, histone H2A variant, H2AX and PARP-1. The increase of γ-H2AX adds to the hypothesis that HnB may be associated with DNA-damaging activity. γ-H2AX plays an essential role in the recruitment and accumulation of DNA repair proteins at sites of double-strand break (DSB) damage due to exposure to DNA-damaging compounds (PAHs and acrylamide), such as those found in the HnB mainstream smoke (Dickey et al., 2009). Poly (ADP-ribose) polymerase (PARP) is a major repair mechanism resolving DNA lesions caused by endogenous processes, including oxidative stress in spermatocytes (Agarwal et al., 2009), and plays a key role in a wide range of testis-associated processes, including maintenance of genomic stability, transcriptional regulation, centromere function and mitotic spindle formation, trafficking of endosomal vesicles, apoptosis, and necrosis (Omar et al., 2018). However, overactivation of PARP-1 represents an important mechanism of tissue damage in various pathological conditions related to oxidative stress, resulting in cell death by apoptosis, which contributes to OS-related infertility (Celik-Ozenci and Tasatargil, 2013). Interestingly, some PAHs, such as benzo(a)pyrene, have been reported to induce necrosis through activation of PARP-1 (Lin et al., 2008), whereas nicotine has been reported to down-regulate the expression of PARP-1 (Lu et al., 2017), reinforcing the hypothesis that, even when present in lower doses compared to tobacco smoke, PAHs in HnB mainstream smoke can contribute to testicular toxicity. Here, no significant changes in the xeroderma pigmentosum group C (XPC) DNA repair protein were observed.

Next, we investigated the effects of HnB exposure on testis antioxidant response and our results indicate activation of NRF2-dependent machinery, including catalase (CAT), superoxide dismutase (SOD), glutathione reductase (GSSG-Red), and NADPH quinone reductase (NQO1), whereas no significant changes were observed for glutathione peroxidase (GSH-Px) in testicular tissue. These findings are in line with recent data from passive smoking animal models (Yao et al., 2022). Xanthine oxidoreductase (XOR) catalyzes the conversion of hypoxanthine to xanthine, and it has been proposed as an oxidative stress marker, since under OS conditions, it can be converted from a dehydrogenase form that uses NADPH as electron acceptor, to an oxidase (XO) form that uses oxygen to generate H_2_O_2_ (Frijhoff et al., 2015). We observed the induction of XO activity in the HnB group, and these data are in line with some evidence indicating that XO represents a critical mediator of DSB induction by cigarette smoke, in particular, XO inhibitors attenuate levels of phosphorylated γ-H2AX in cigarette smoking rodent models (Kim et al., 2013).

From the available literature, the role of sirtuins in the signaling of oxidative stress and their antioxidant defense in the cell emerges (Cheng et al., 2014). SIRT-1 has been called the “sensor” and “guardian of the redox state” and it is normally overexpressed following OS injuries; on the other hand, the SIRT-1 downregulation can be attributed to competitive consumption of NAD^+^ caused by DNA damage-induced PARP-1 activation (Zhang et al., 2021). Testicular tissue is highly vulnerable to OS and SIRT1 has also been found to have a significant role in the male reproductive system as it is involved in spermatogenesis by influencing specific functions of male germ cells, Sertoli cells, and Leydig cells (Alam et al., 2021).

Although the mechanism of inflammation-mediated male reproductive failure has not yet been fully elucidated, numerous studies indicate that increased production of pro-inflammatory mediators such as ROS, interleukins and NO can lead to a reduction in steroidogenic function (Dutta et al., 2021; Azenabor et al., 2015). Several investigations have shown that ROS can cause inflammation by activating the transcription factor NF-kB, which activates innate immune responses generating proinflammatory cytokines, such as IL-1, IL-6. In addition, the Sertoli cells can release many endogenous inflammatory mediators, including TNF-α (Luo et al., 2018; Wardyn et al., 2015). Our model shows a significant increase of NF-kB target genes TNF-α, IL-1β, IL-6 in testis samples from exposed animals, while no changes in IL-8 expression were noted. Low levels of serum androgens have been reported to correlate with increased expression of the circulating pro-inflammatory cytokines IL-1β, IL6 and TNF-α which is reported to contribute to the decrease of testosterone production via Toll-like-Receptor (TLR) pathway activation (Samir et al., 2017; Matzkin et al., 2016). Furthermore, DNA base alterations caused by ROS may contribute to inflammation as base excision repair by OGG1, resulting in the activation of the NF-kB pathway, inducing expression of pro-inflammatory genes and accumulation of inflammatory cells (Visnes et al., 2018). Both TNF-α and IL-1 β are associated with reduced sperm count, sperm motility and morphology, as well as increased ROS and higher MDA levels (Abdelzaher et al., 2022; Di Persio et al., 2021; Jaiswal et al., 2013). The increasing trend of COX-2 expression, although not of statistical significance, may be of interest considering that it is a marker of testicular tumour promotion, progression, and metastasis (Matsuyama and Yoshimura, 2009; Petricca et al., 2023), and a positive correlation between IL-1β levels and COX-2 expression has been described in the testes of infertile patients (Matzkin et al., 2010; Guazzone et al., 2009). The present study lacks an assessment of immune cell changes. These data would have provided interesting insights into the pro-inflammatory effects discussed above.

Consistently with the findings discussed above, androgenic enzyme data indicate downregulation of testicular 3β-HSD and 17β-HSD, two key enzymes of steroidogenesis whose impairment is recognized as a marker of testicular failure (Ullah et al., 2018). Similar changes have been reported in rodent models of tobacco smoking, e-cigarette and B(a)P exposure (Vivarelli et al., 2019b; Jana et al., 2010; Dechanet et al., 2011; Banerjee et al., 2016), leading to decreased testosterone biosynthesis, sperm apoptosis, along with decreased plasma testosterone and impaired steroidogenesis of Leydig cells. Our model confirms a significant reduction in plasma testosterone levels in exposed animals. Interestingly, the decrease in sorbitol dehydrogenase (SDH), which plays a key role in the maturation of the germinal epithelial layer of the seminiferous tubule, combined with the increase in lactate dehydrogenase (LDH), supports the hypothesis of damage to the germinal epithelium (Alboghobeish et al., 2019).

Since exposure to PAHs and tobacco smoke can alter the cell cycle by inducing apoptosis in the testis cells (Xu et al., 2019), we explored putative changes in the whole expression of Bax and Bcl-2 as markers of apoptotic/anti-apoptotic homeostasis. Our results show no significant changes in the Bax/Bcl-2 ratio and no changes in p-38 activation, in contrast to data from smoking and passive smoking models (Tommasi et al., 2022; Dey et al., 2011). On the other hand, animals exposed to HnB smoke showed significant activation of (ERK)1/2 mitogen-activated protein (MAP) kinase pathways involved in Sertoli cell proliferation and normal spermatogenesis (Tommasi et al., 2022), and a marked increase in c-MYC expression as a marker of tumour-promoting potential as reported in smoking models (Dey et al., 2011). These data suggest a possible impairment of the spermatogenesis.

Since PAHs are aryl hydrocarbon receptor (AhR) ligands that exert their toxic effects on both male and female reproductive systems, we studied the effects of HnB smoking on cytochrome P450 (CYP) superfamily in testicular tissue. As expected, exposure to HnB mainstream led to a significant induction of CYP1A1, including protein expression, CYP2B1/2 (activating olefins and halogenated hydrocarbons) and CYP2A1/2 (activating, polychlorinated biphenyls, aromatic amines, PAHs, and alkylnitrosamines). Similar findings are usually observed in the liver from tobacco smoke rodent models (Yue et al., 2009; Mori et al., 2003), as well as in rat testis after exposure to B(a)P (Jana et al., 2010; Banerjee et al., 2016), and more recently have also been reported in lung tissue from an *in vivo* model of HnB (Vivarelli et al., 2022). These enzymes are responsible for the metabolism of B(a)P to a carcinogenic compound BPDE [benzo(a)pyrene-7,8-diol-9,10-epoxide], and CYP induction is associated with damage to the DNA of spermatozoa and ROS overexpression (Revel et al., 2001), as here observed.

Overall, the results presented here show how the use of “Modified Risk Tobacco Products” can induce some of the same toxicological outcomes triggered by tobacco smoking, although less marked. Considering the limited exposure time in this study (4 weeks), it is highly unlikely any modifications in testicular physiology will impact on the histology or fertility (sperm count) of these animals. For this reason, we focused our study on organ physiology only. Longer exposure studies are needed to analyze changes in testicular histology and controlled clinical studies will help to clarify the impact of “Modified Risk Tobacco Products” on humans.

**FIGURE 8:**
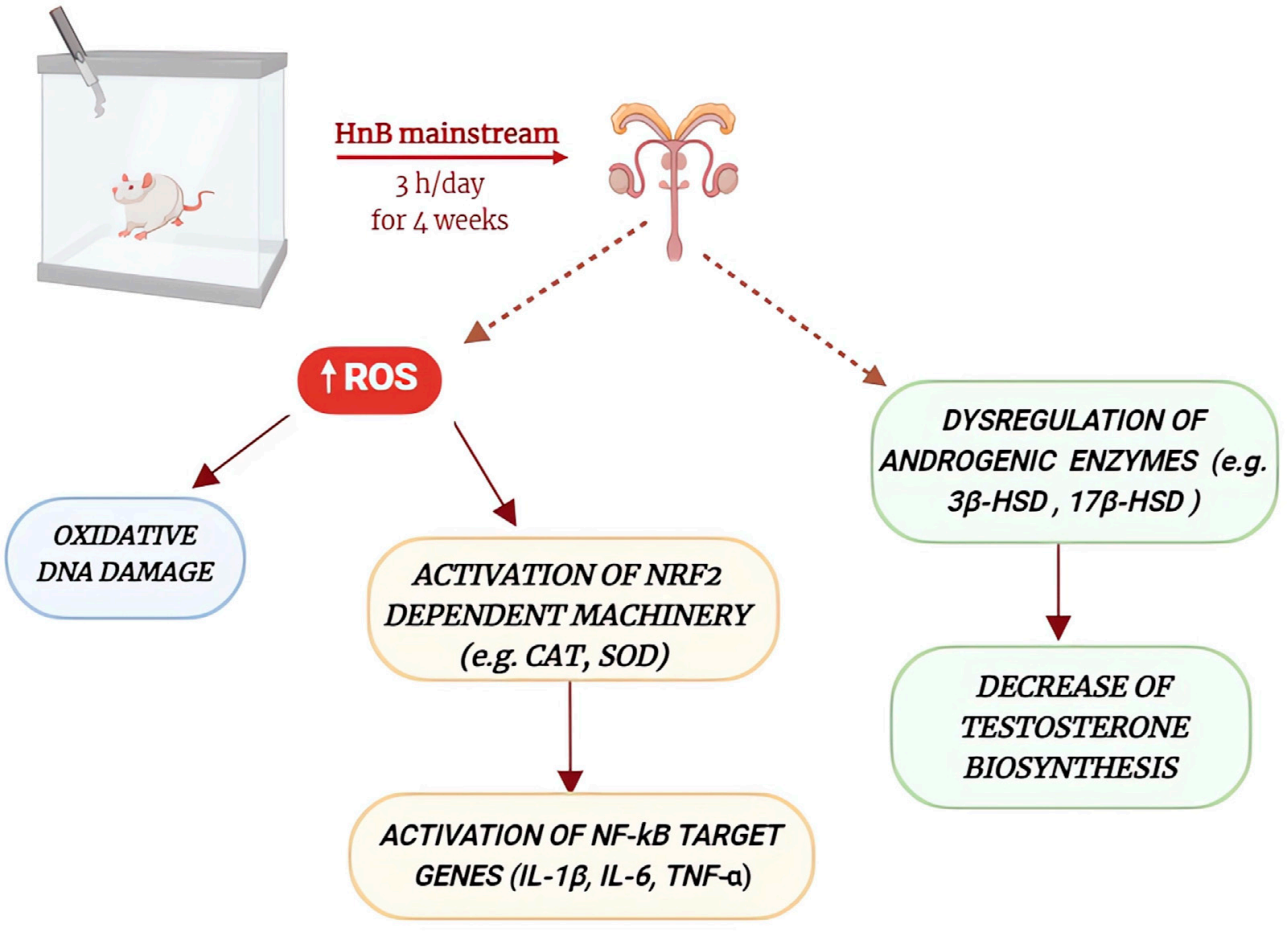
Schematic representation of the main effects of HnB mainstream exposure on rat testicular function. HnB smoke exposure increases the production of reactive oxygen species, leading to oxidative DNA damage and the activation of DNA repair enzymes, as well as the activation of NRF2 and NF-kB signaling pathways. These alterations, together with the dysregulation of some androgenic enzymes and the decrease of testosterone biosynthesis, may contribute to the impairment of testicular function.

The present study shows how the HnB mainstream releases aldehydes and PAHs indicative of thermal degradation and incomplete combustion of tobacco, raising questions as to whether HnB devices using tobacco generate smoke rather than aerosol, as some regulatory agencies and media have reported. Overall, the results presented here indicate that animals exposed to HnB smoke show higher levels of oxidative stress markers, including those associated with DNA damage, as well as higher levels of pro-inflammatory cytokines. Furthermore, the impairment of some androgenic key enzymes and those related to the activity of seminiferous epithelium, together with the decrease in testosterone levels, could suggest an impairment of gonadal function through the alteration of some cellular pathways typically associated with tobacco consumption (Figure 8). However, since here no specific tests on testosterone release at the testicular level were performed, it cannot be definitively assumed that this phenomenon is directly related to HnB exposure on gonadal function. Further investigations are needed to better clarify this point, which is certainly of public health interest. Our data, albeit preliminary, indicates that although present in lower concentration in comparison to those recorded in best-selling brands of cigarettes, the mixture of toxic elements released by HnB devices could pose a risk for people using this new method for tobacco consumption. On the other hand, HnB may fail to offer the risk reduction that is at the heart of their popularity. More studies are needed to further explore testicular damage at a cellular level, but our data calls for caution when promoting HnB products as a part of smoking cessation efforts.

## 5 Limitations of the study

Below, some limitations of the study will be discussed in order to allow for a more accurate interpretation and contextualization of the presented results.

The present study does not include histological image acquisition, nor a sperm count or morphology tests that are needed to fully to define the magnitude of HnB exposure on the spermatogenesis, especially considering that sperm adverse outcomes can occur as a result of short-term exposure (Darmishonnejad et al., 2024). Protein expression experiments were run in duplicate independently by different personnel Furthermore, the data presented here come from an experimental whole-body exposure setup, and the application of different exposure modalities (whole-body or nose-only) involve some differences that could influence the results. However, comparative experiments indicate that the respiratory disease endpoints are established in both exposure systems. Nose-only exposure to cigarette smoke resulted in significantly stronger effects than whole-body exposure in terms of epithelial degeneration and ulceration in nasal epithelia, lung inflammation, and it is associated to a higher stress response compared to whole-body mode (Kogel et al., 2021). The higher toxicity can possibly be explained by the higher local exposure that occurred in nose-only mode, on the other hand, in whole-body exposure, the inhaled dose could be lower due to the deposition losses of constituents on the fur and the larger contact surfaces of the chamber (Pauluhn and Mohr, 2000). It is finally necessary to point out that the effects investigated in the present study are only compared with the negative control group. The absence of an experimental unit exposed to substances known to alter, within well-established limits, the investigated parameters would have allowed for a better appreciation of the magnitude of such changes and facilitated a more accurate extrapolation of the results.

## Data Availability

The original contributions presented in the study are included in the article/Supplementary Material, further inquiries can be directed to the corresponding authors.
