# Supplementary material for: Heat-not-burn technology affects plasma testosterone levels and markers of inflammation, oxidative stress in the testes of rats
Source: Front Toxicol. 2025 Jan 20;6:1515850. doi: 10.3389/ftox.2024.1515850 (PMC11788375; doi:10.3389/ftox.2024.1515850)

Blot 1 Lines 1-4 CTRL; Lines 5-9 HnB
NRF2

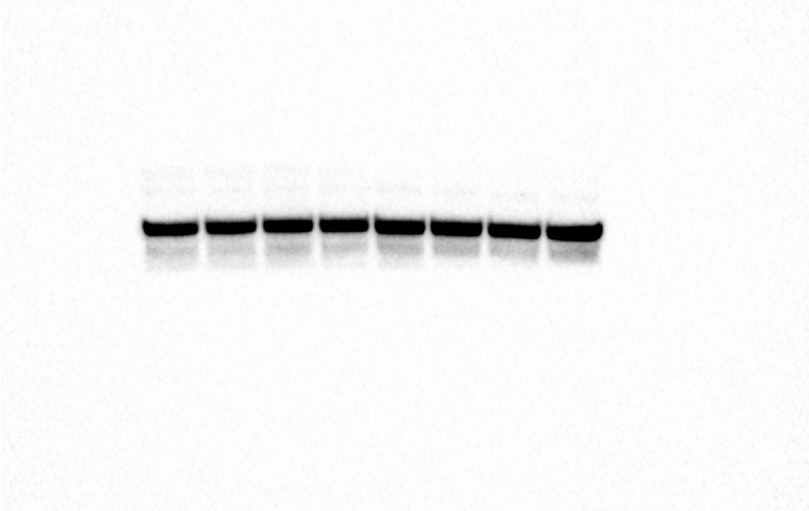

Blot 2 Lines 1-4 CTRL; Lines 5-9 HnB
NRF2

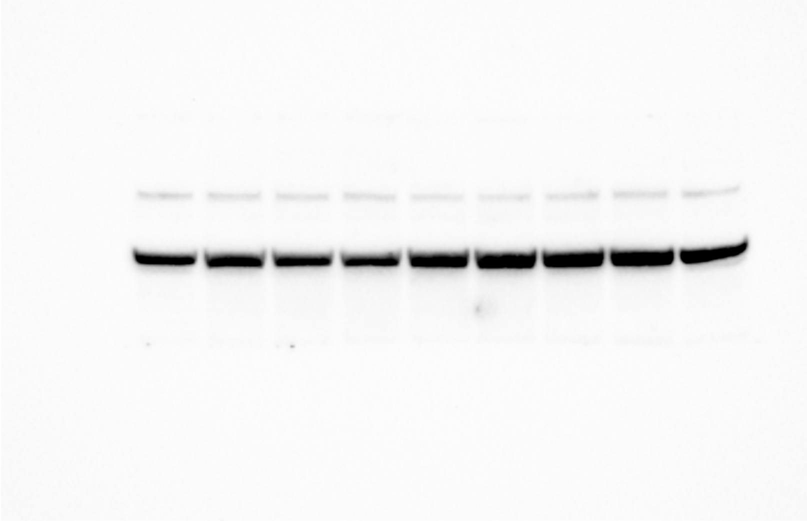

Blot 1 Lines 1-4 CTRL; Lines 5-9 HnB
a-tubulin

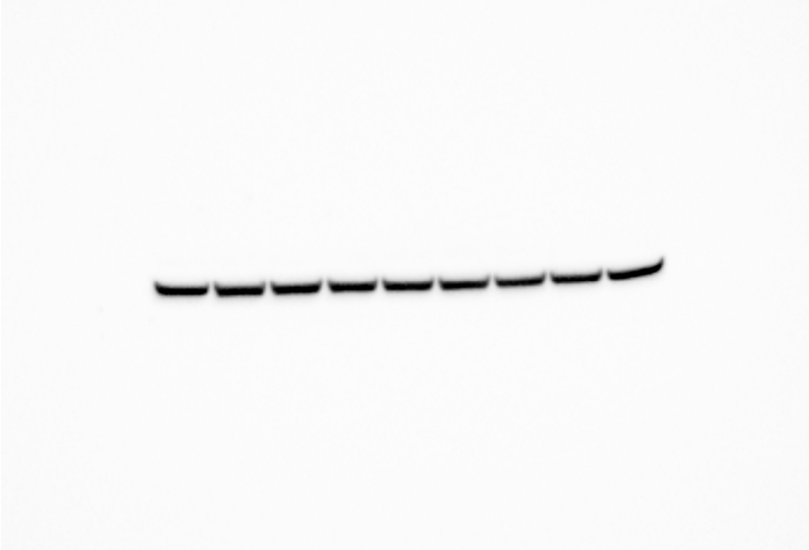

Blot 2 Lines 1-4 CTRL; Lines 5-9 HnB
a-tubulin

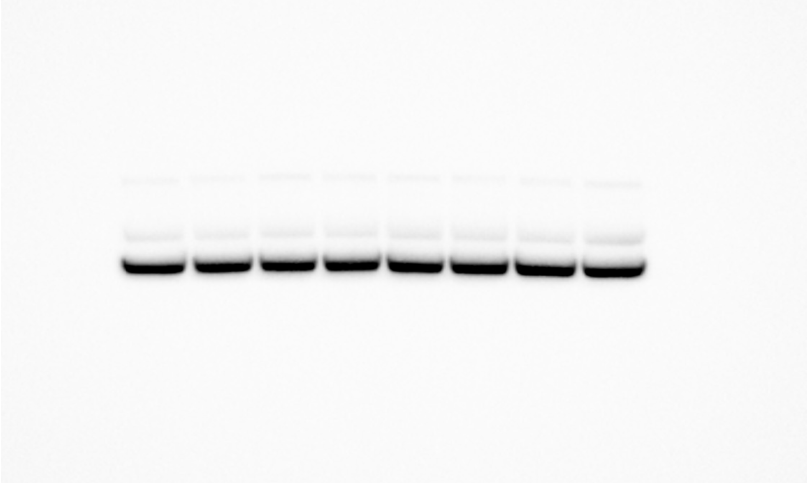

Blot 1 Lines 1-4 CTRL; Lines 5-9 HnB
SOD-1

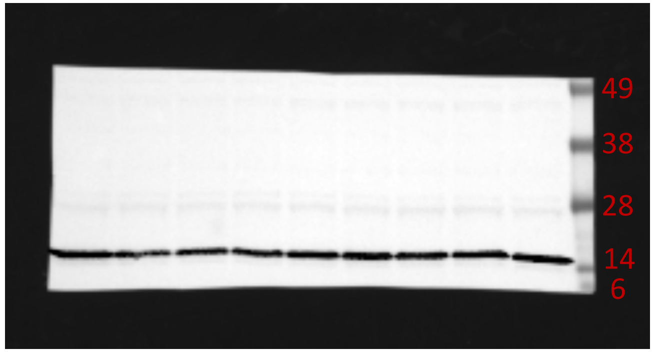

Blot 2 Lines 1-4 CTRL; Lines 5-9 HnB
SOD-1

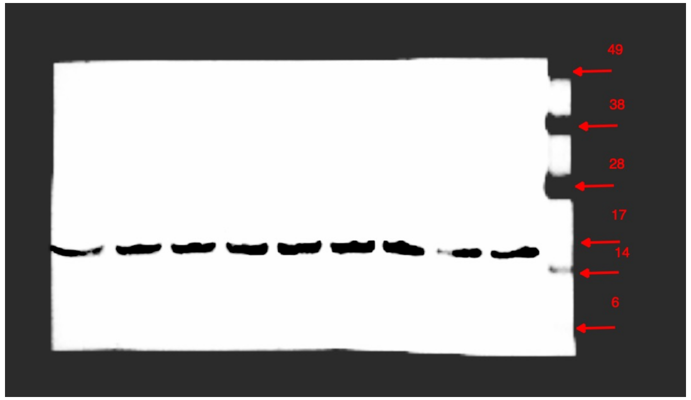

Blot 1 Lines 1-4 CTRL; Lines 5-9 HnB
a-tubulin

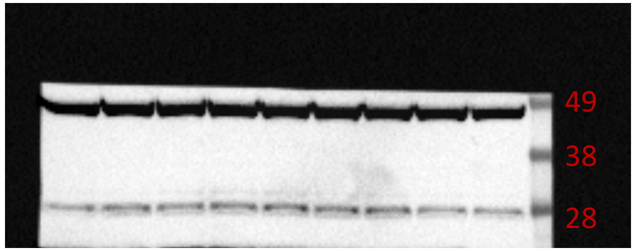

Blot 2 Lines 1-4 CTRL; Lines 5-9 HnB
a-tubulin

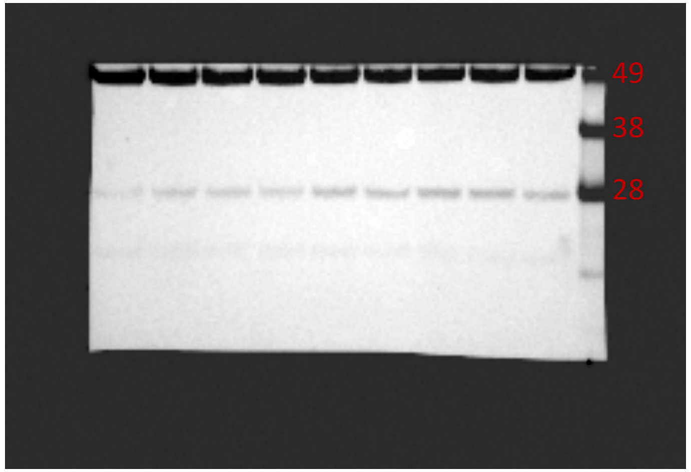

Blot 1 Lines 1-4 CTRL; Lines 5-9 HnB
SIRT-1

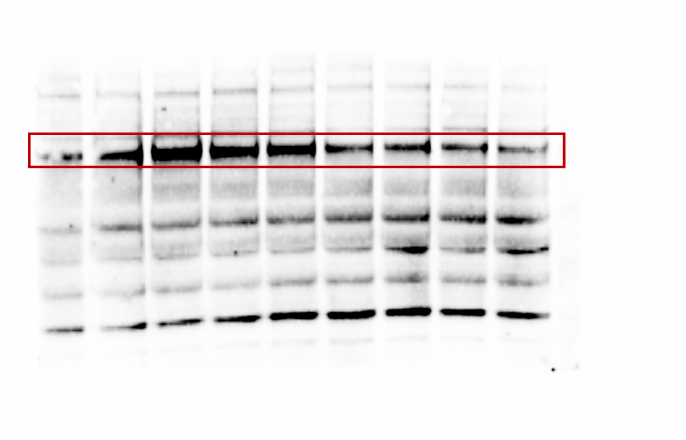

Blot 2 Lines 1-4 CTRL; Lines 5-9 HnB
SIRT-1

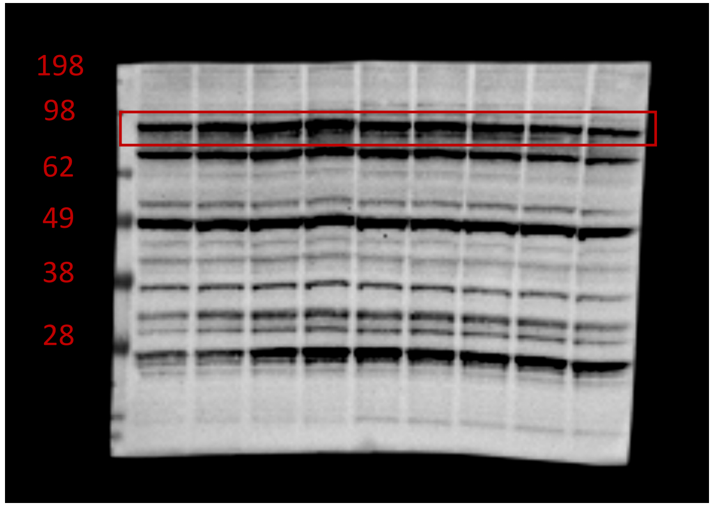

Blot 1 Lines 1-4 CTRL; Lines 5-9 HnB
a-tubulin

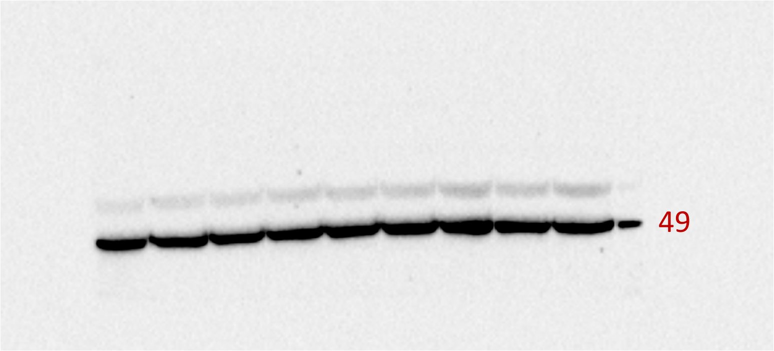

Blot 2 Lines 1-4 CTRL; Lines 5-9 HnB
a-tubulin

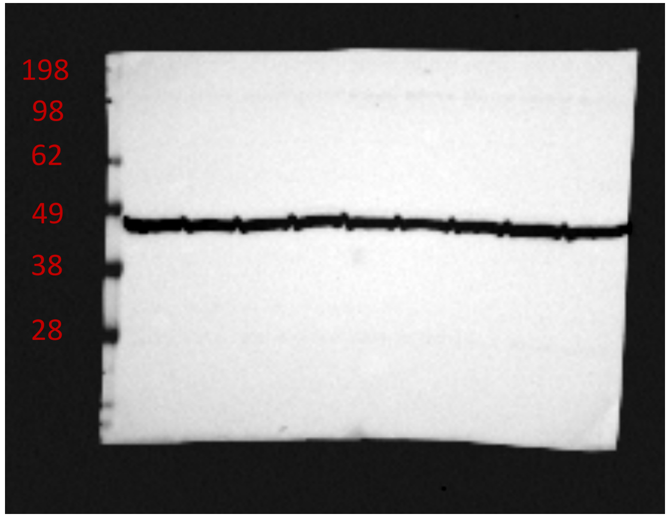

Blot 1 Lines 1-4 CTRL; Lines 5-8 HnB
p-NF-kB

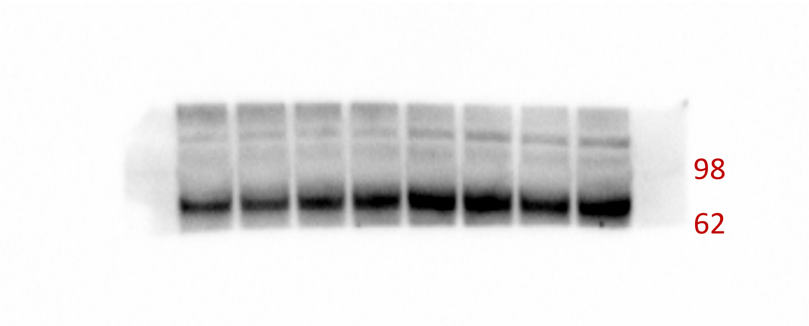

Blot 2 Lines 1-4 CTRL; Lines 5-9 HnB
p-NF-kB

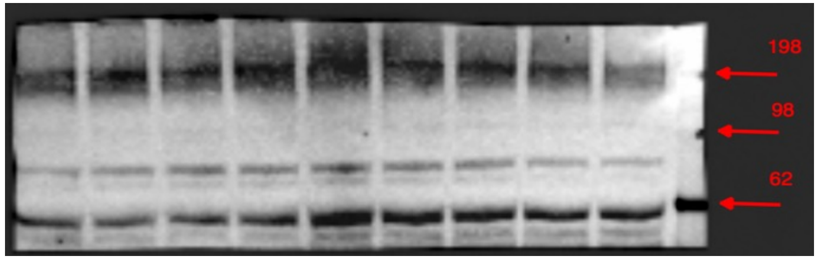

Blot 1 Lines 1-4 CTRL; Lines 5-8 HnB
Tot-NF-kB

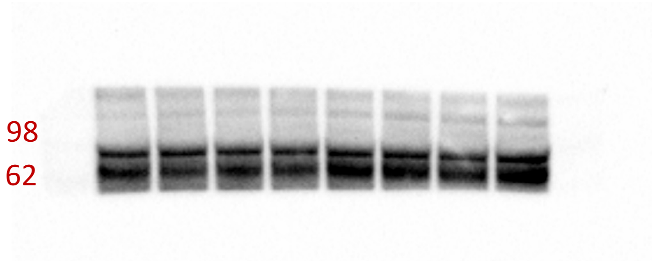

Blot 1 Lines 1-4 CTRL; Lines 5-9 HnB
Tot-NF-kB

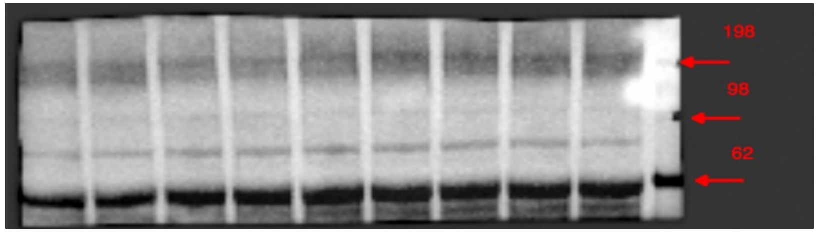

Blot 1 Lines 1-4 CTRL; Lines 5-8 HnB
a-tubulin

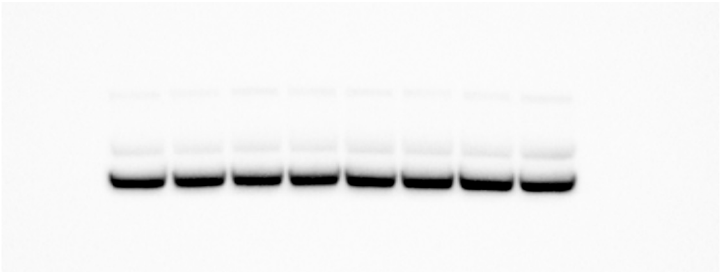

Blot 2 Lines 1-4 CTRL; Lines 5-9 HnB
a-tubulin

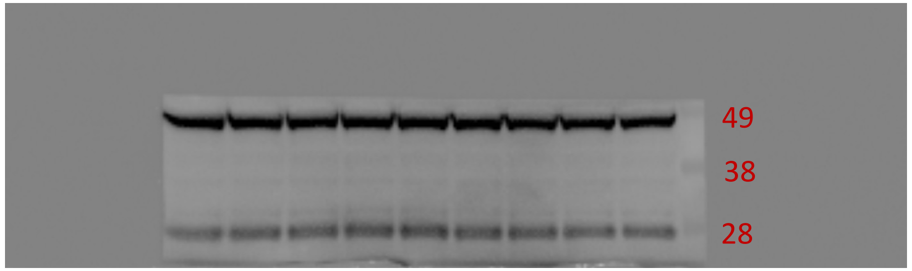

Blot 1 Lines 1-4 CTRL; Lines 5-9 HnB
IL-1b

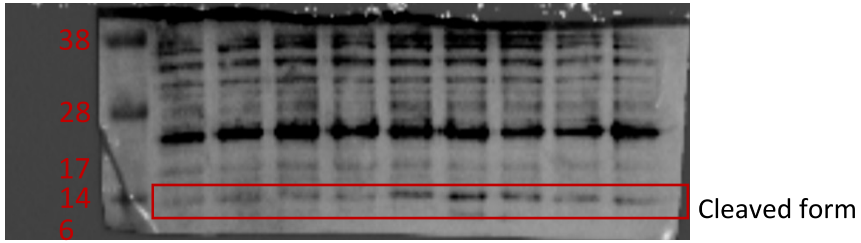

Blot 2 Lines 1-4 CTRL; Lines 5-9 HnB
IL-1b

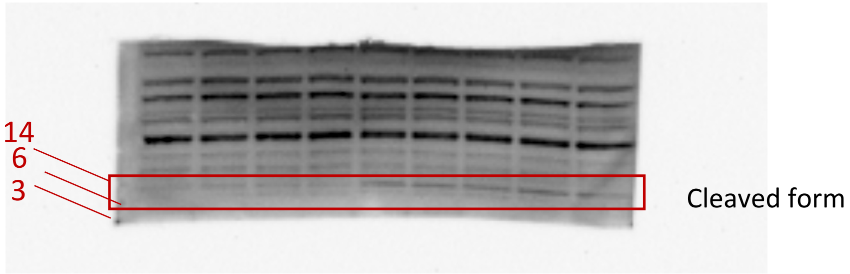

Blot 2 Lines 1-4 CTRL; Lines 5-9 HnB
a-tubulin

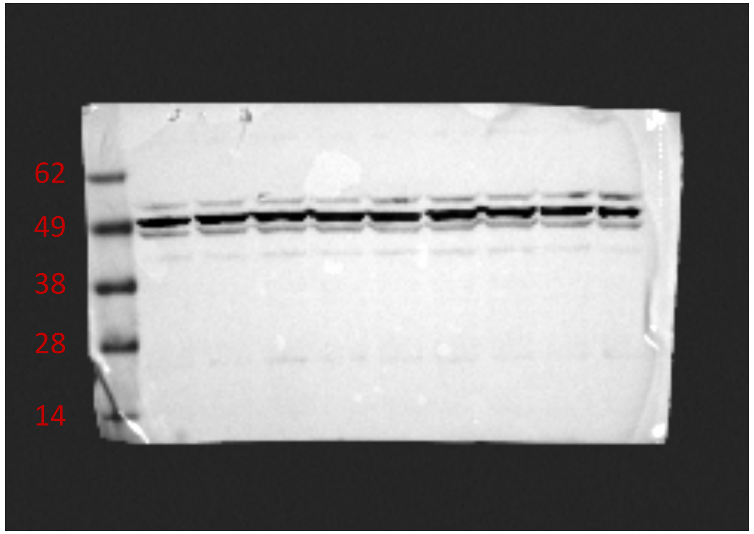

Blot 2 Lines 1-4 CTRL; Lines 5-9 HnB
a-tubulin

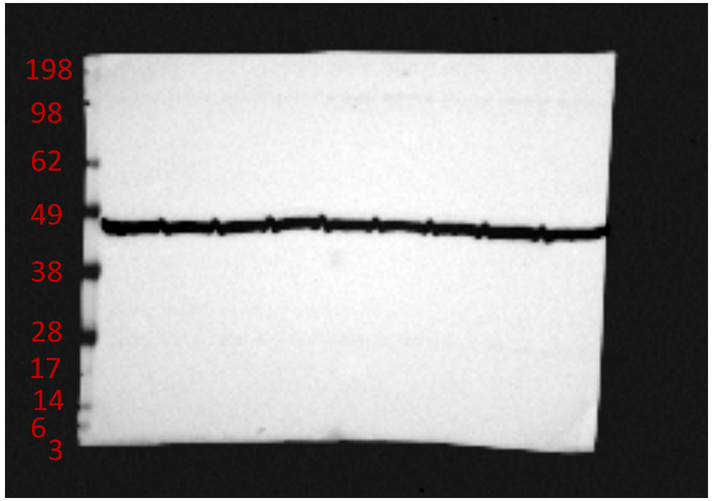

Blot 1 Lines 1-4 CTRL; Lines 5-9 HnB
IL-6

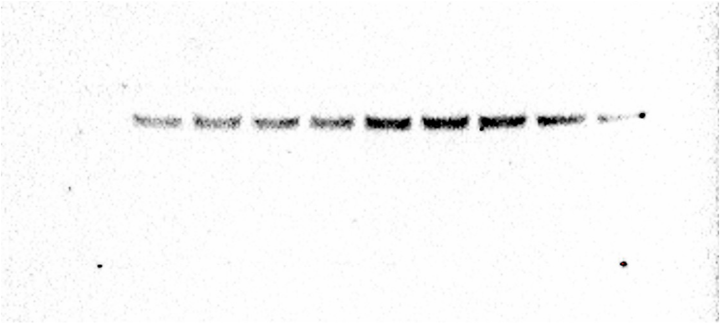

Blot 2 Lines 1-4 CTRL; Lines 5-9 HnB
IL-6

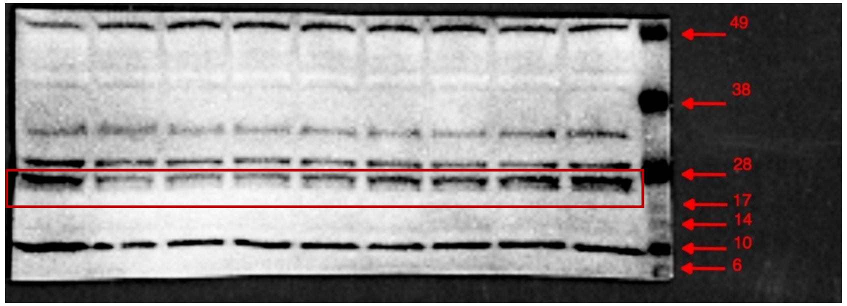

Blot 2 Lines 1-4 CTRL; Lines 5-9 HnB
a-tubulin

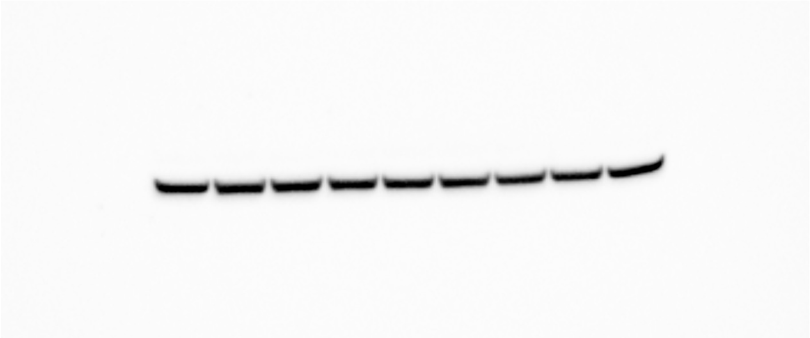

Blot 2 Lines 1-4 CTRL; Lines 5-9 HnB
a-tubulin

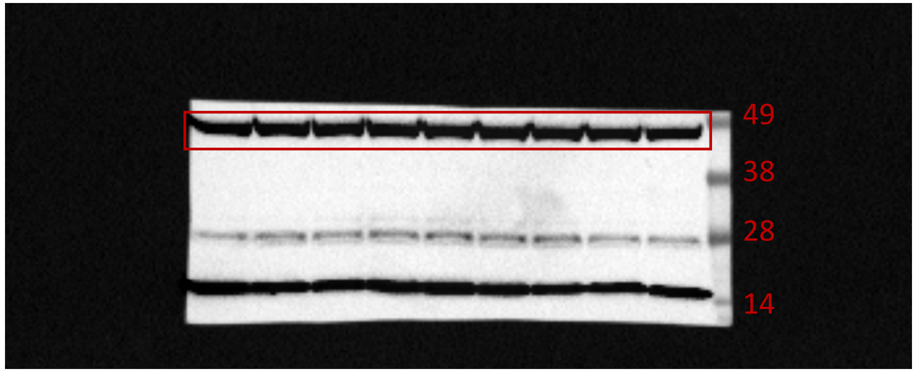

Blot 1 Lines 1-4 CTRL; Lines 5-9 HnB
IL-8

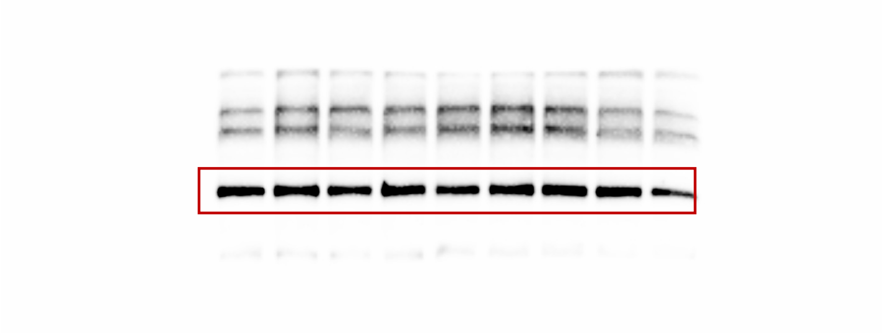

Blot 2 Lines 1-4 CTRL; Lines 5-9 HnB
IL-6

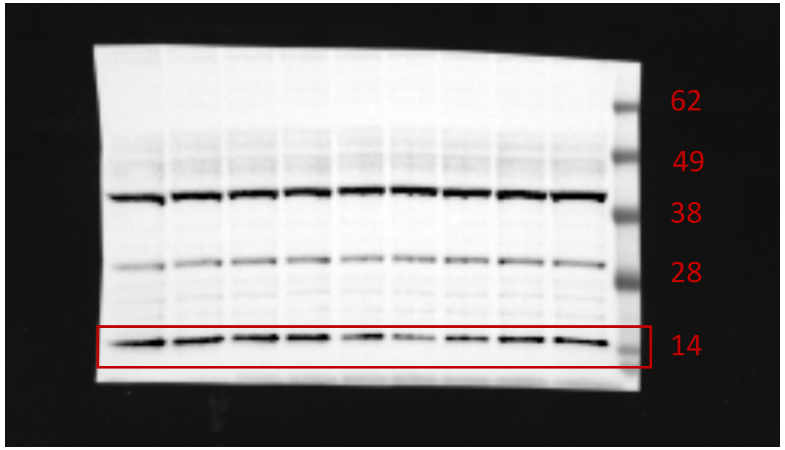

Blot 2 Lines 1-4 CTRL; Lines 5-9 HnB
a-tubulin

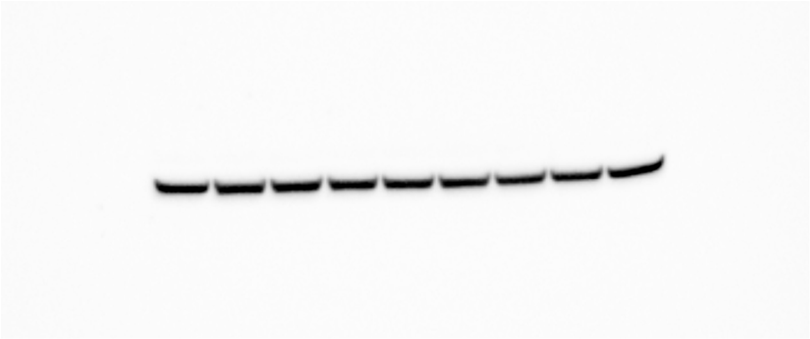

Blot 2 Lines 1-4 CTRL; Lines 5-9 HnB
a-tubulin

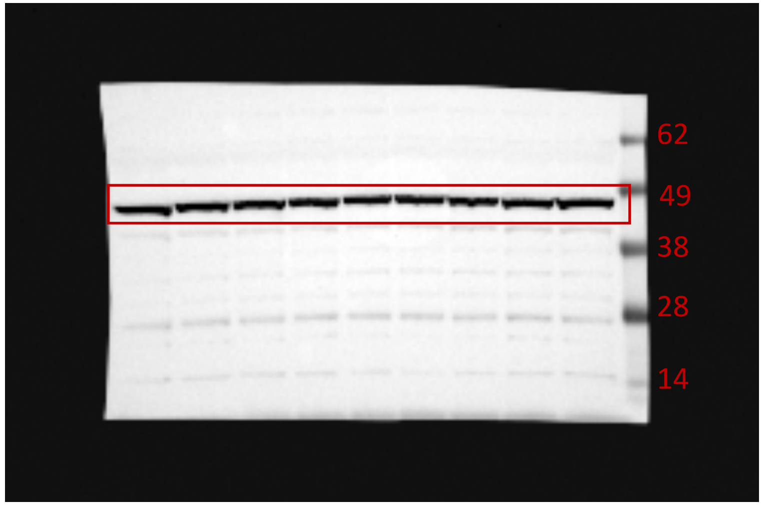

Blot 1 Lines 1-4 CTRL; Lines 5-9 HnB
p-ERK 1/2

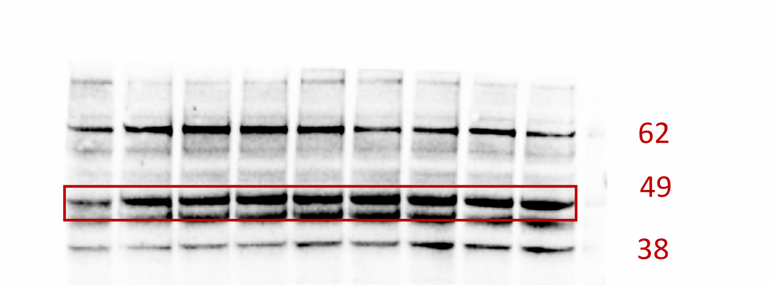

Blot 1 Lines 1-4 CTRL; Lines 5-9 HnB
Tot-ERK1/2

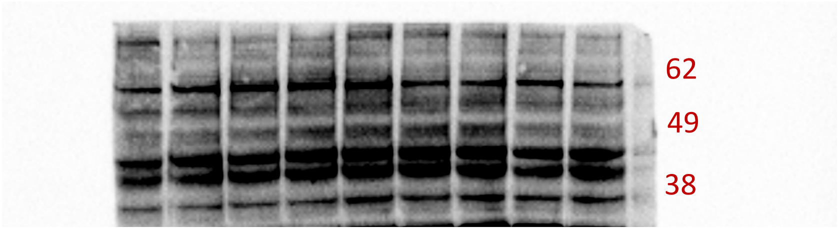

Blot 1 Lines 1-4 CTRL; Lines 5-9 HnB
a-tubulin

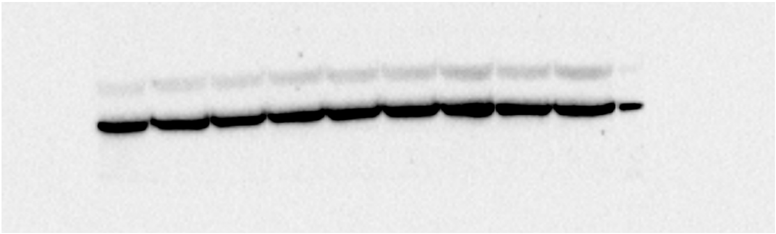

Blot 2 Lines 1-4 CTRL; Lines 5-9 HnB
p-ERK 1/2

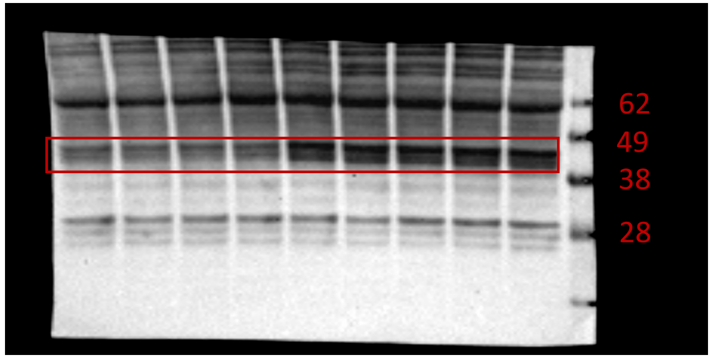

Blot 1 Lines 1-4 CTRL; Lines 5-9 HnB
Tot-ERK 1/2

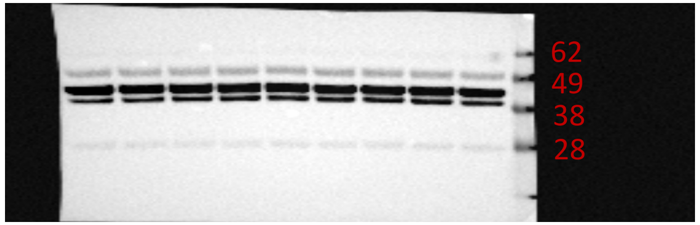

Blot 2 Lines 1-4 CTRL; Lines 5-9 HnB
a-tubulin

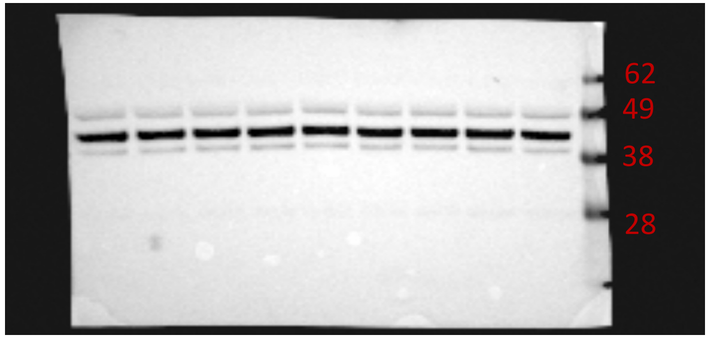

Blot 1 Lines 1-4 CTRL; Lines 5-9 HnB
PARP-1

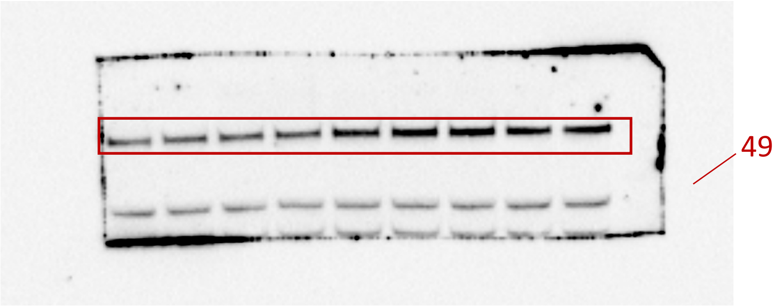

Blot 2 Lines 1-4 CTRL; Lines 5-9 HnB
PARP-1

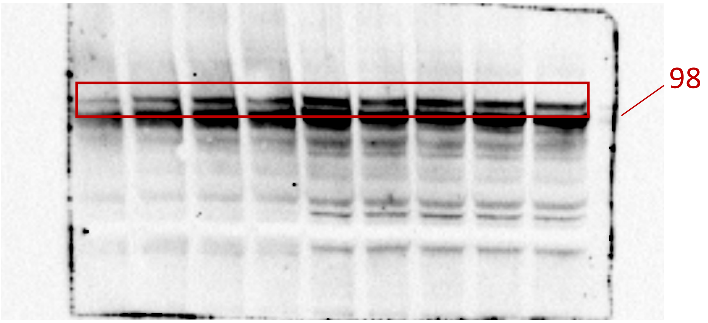

Blot 1 Lines 1-4 CTRL; Lines 5-9 HnB
a-tubulin

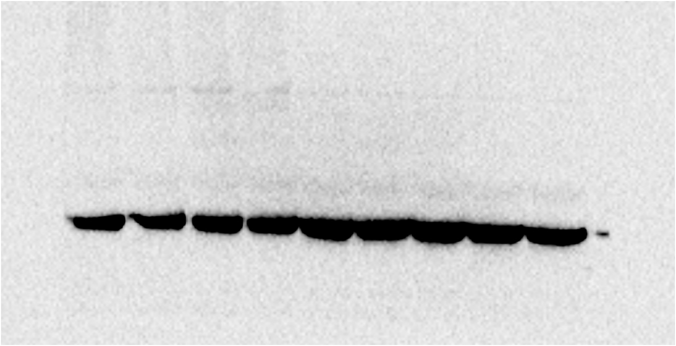

Blot 2 Lines 1-4 CTRL; Lines 5-9 HnB
a-tubulin

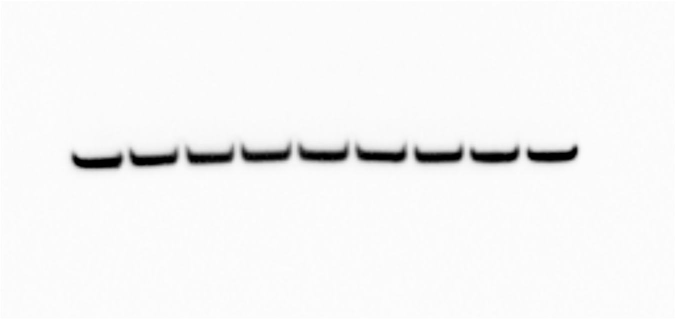

Blot 1 Lines 1-4 CTRL; Lines 5-9 HnB
OGG-1

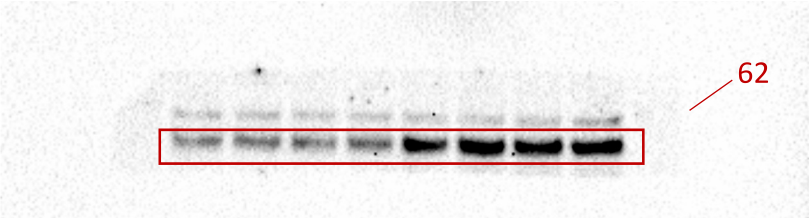

Blot 2 Lines 1-4 CTRL; Lines 5-9 HnB
OGG-1

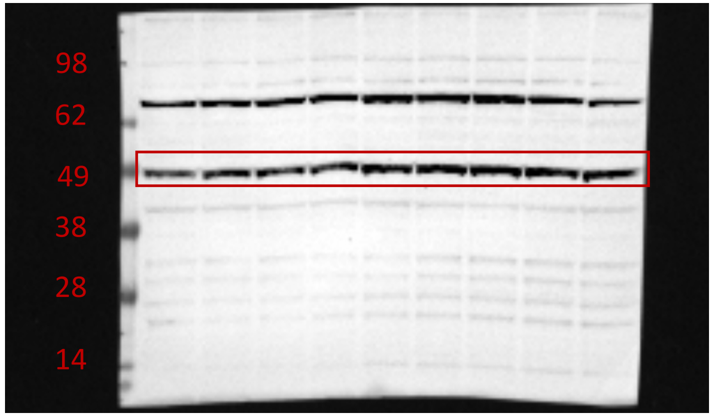

Blot 1 Lines 1-4 CTRL; Lines 5-9 HnB
a-tubulin

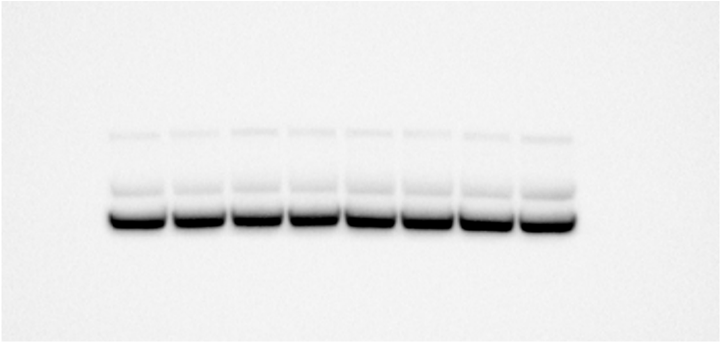

Blot 2 Lines 1-4 CTRL; Lines 4-5 HnB
a-tubulin

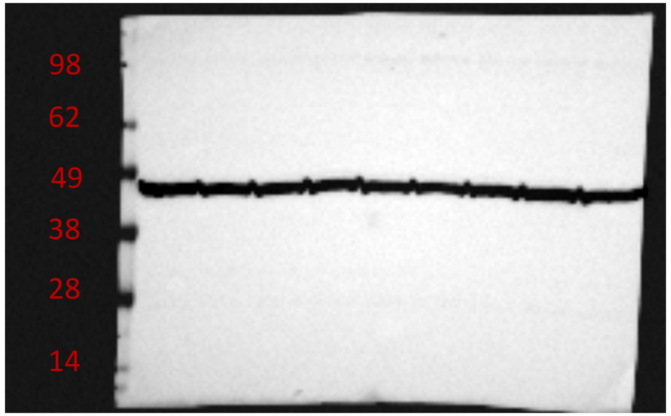

Blot 1 Lines 1-4 CTRL; Lines 5-9 HnB
XPC

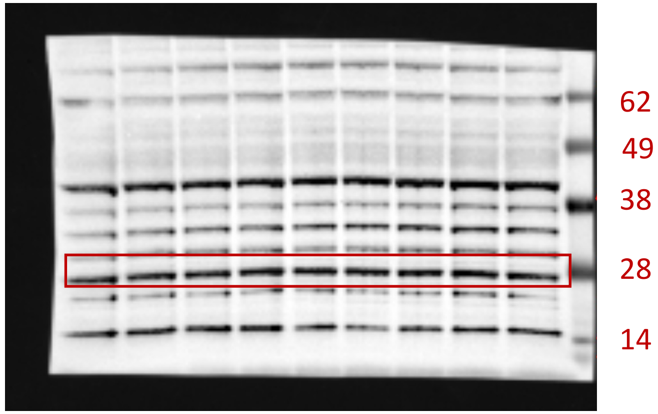

Blot 2 Lines 1-4 CTRL; Lines 5-9 HnB
XPC

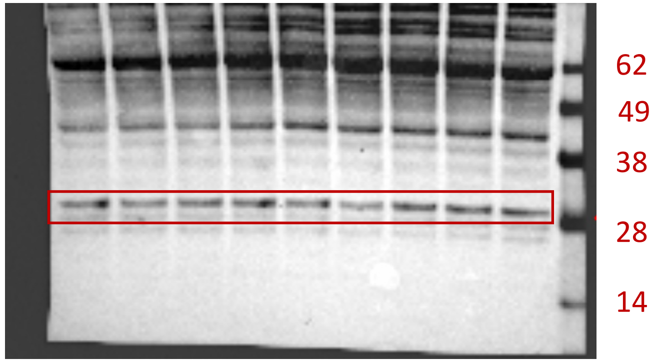

Blot 1 Lines 1-4 CTRL; Lines 5-9 HnB
a-tubulin

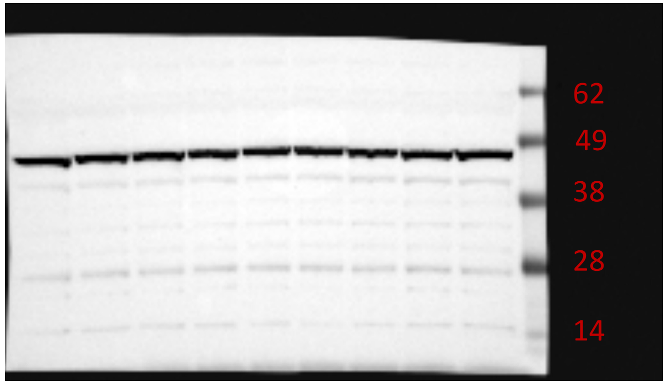

Blot 2 Lines 1-4 CTRL; Lines 5-9 HnB
a-tubulin

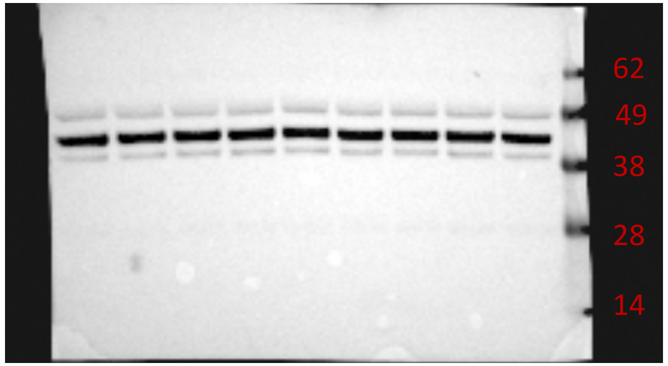

Blot 1 Lines 1-4 CTRL; Lines 5-9 HnB
p-H2AX

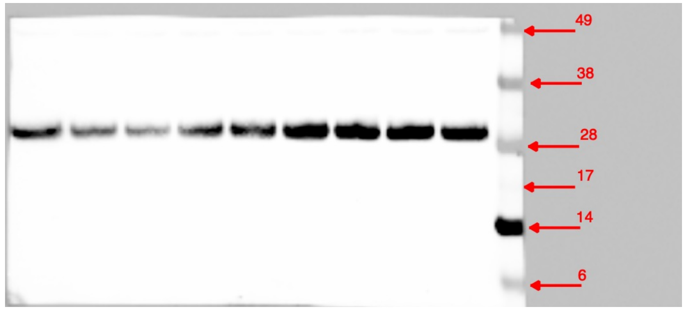

Blot 1 Lines 1-4 CTRL; Lines 5-9 HnB
Tot-H2AX

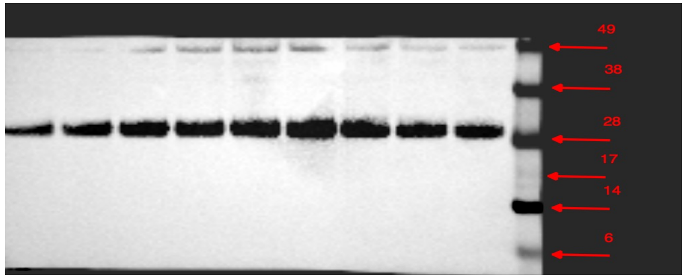

Blot 1 Lines 1-4 CTRL; Lines 5-9 HnB
a-tubulin

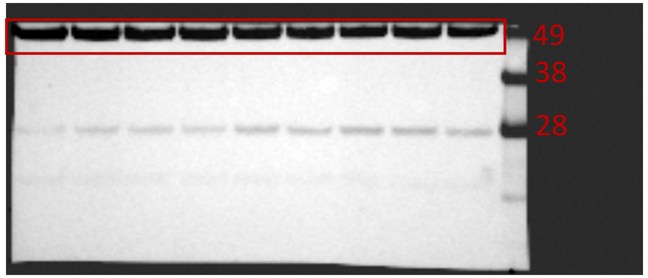

Blot 2 Lines 1-4 CTRL; Lines 5-9 HnB
p-H2AX

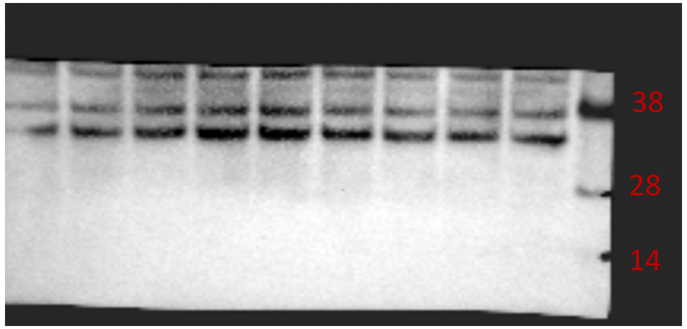

Blot 1 Lines 1-4 CTRL; Lines 5-9 HnB
Tot-H2AX

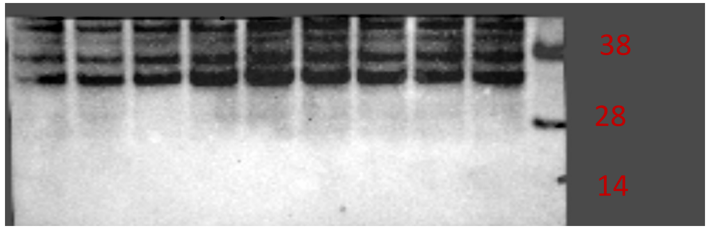

Blot 2 Lines 1-4 CTRL; Lines 5-9 HnB
a-tubulin

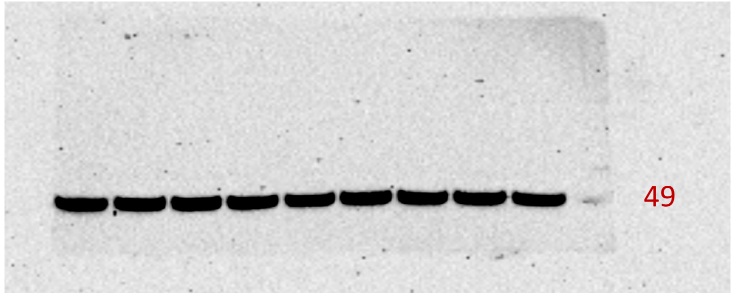

Blot 1 Lines 1-4 CTRL; Lines 5-9 HnB
TNF-a

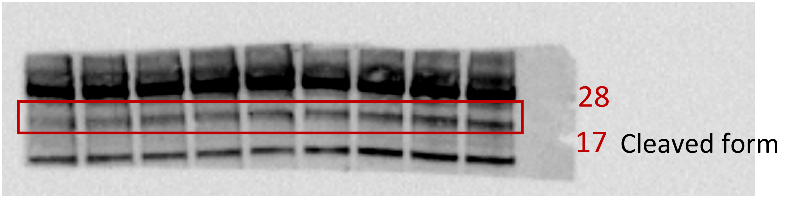

Blot 2 Lines 1-4 CTRL; Lines 5-9 HnB
TNF-a

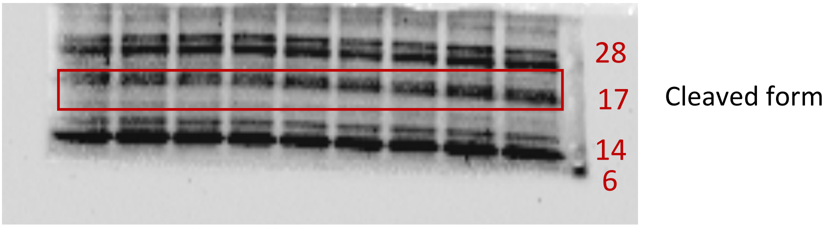

Blot 1 Lines 1-4 CTRL; Lines 5-9 HnB
a-tubulin

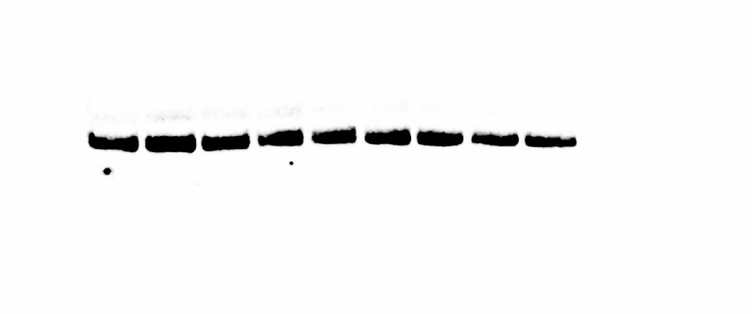

Blot 2 Lines 1-4 CTRL; Lines 5-9 HnB
a-tubulin

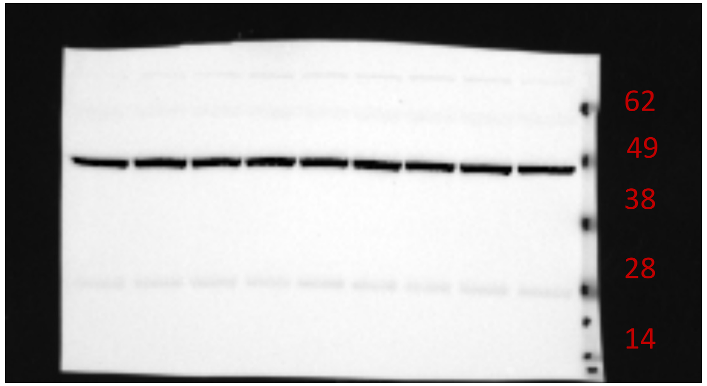

Blot 1 Lines 1-4 CTRL; Lines 5-8 HnB
Bax

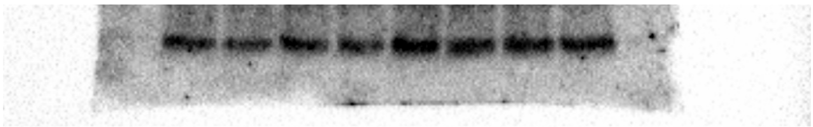

Blot 2 Lines 1-4 CTRL; Lines 5-9 HnB
Bax

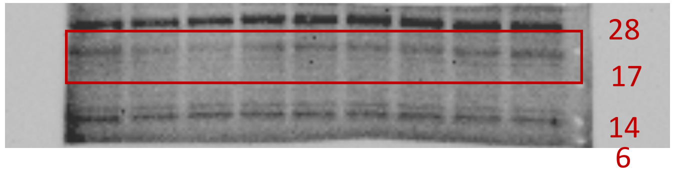

Blot 1 Lines 1-4 CTRL; Lines 5-8 HnB
a-tubulin

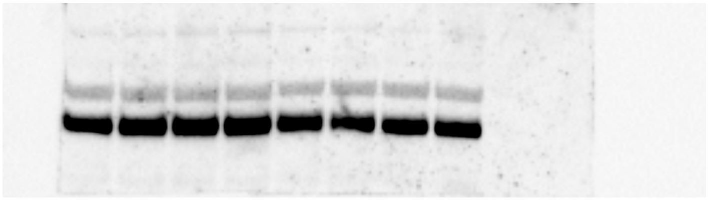

Blot 2 Lines 1-4 CTRL; Lines 5-9 HnB
a-tubulin

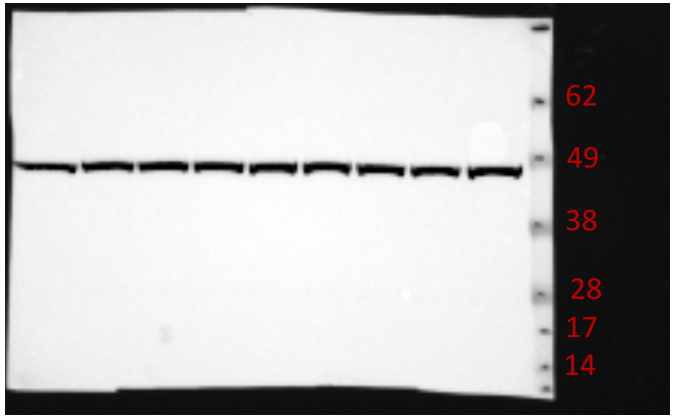

Blot 1 Lines 1-4 CTRL; Lines 5-8 HnB
Bcl-2

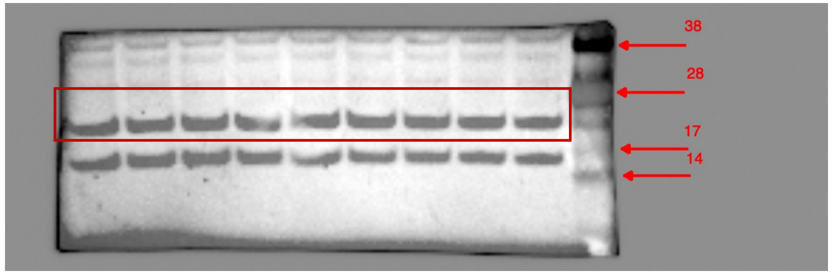

Blot 2 Lines 1-4 CTRL; Lines 5-9HnB
Bcl-2

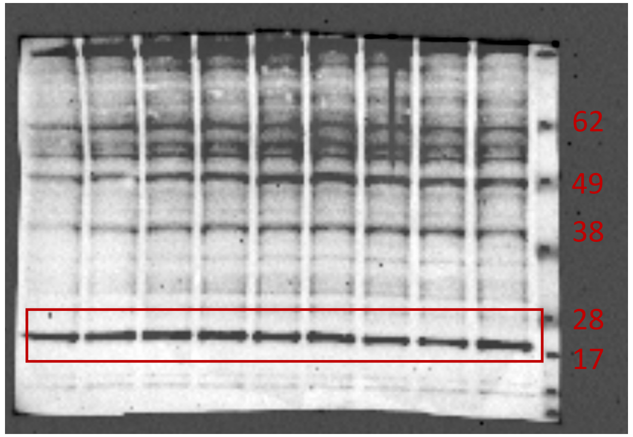

Blot 1 Lines 1-4 CTRL; Lines 5-8 HnB
a-tubulin

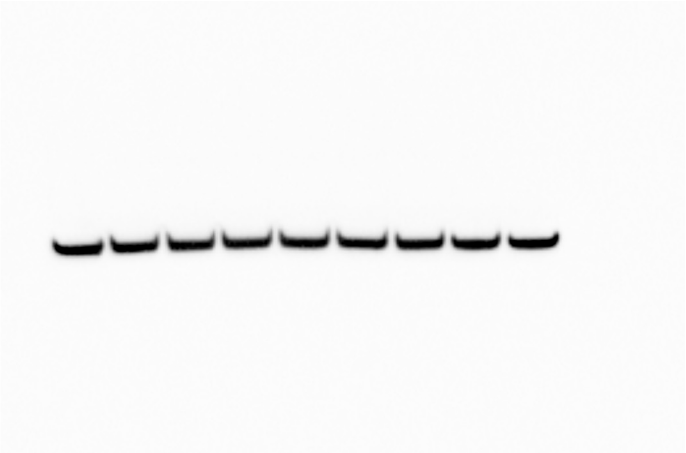

Blot 2 Lines 1-4 CTRL; Lines 5-9 HnB
a-tubulin

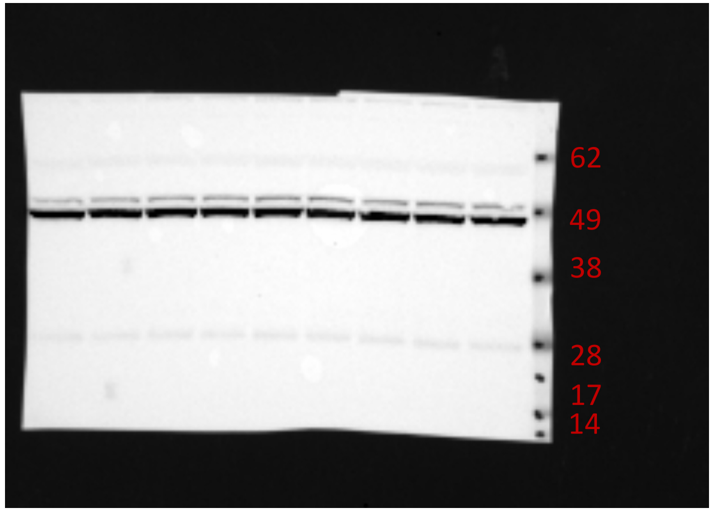

Blot 1 Lines 1-5 HnB; Lines 6-9 CTRL
COX-2

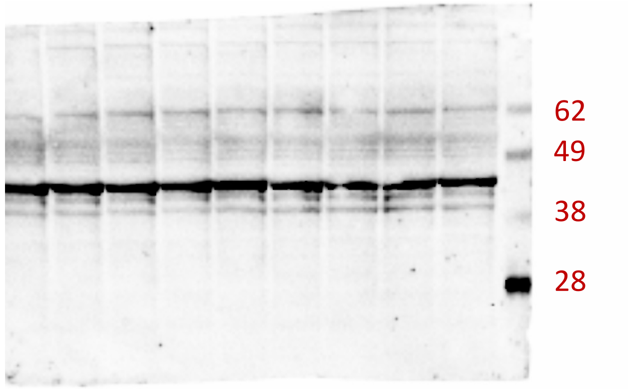

Blot 2 Lines 1-4 CTRL; Lines 5-9 HnB
COX-2

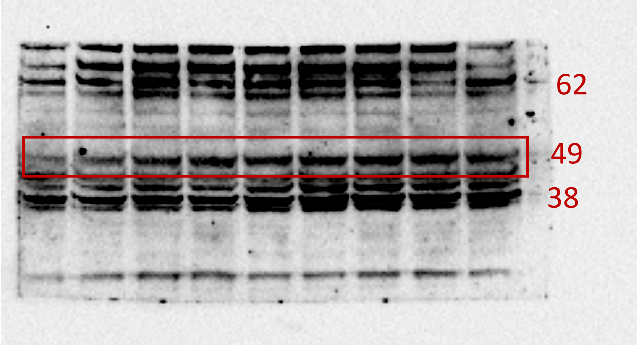

Blot 1 Lines 1-5 HnB; Lines 6-9 HnB
a-tubulin

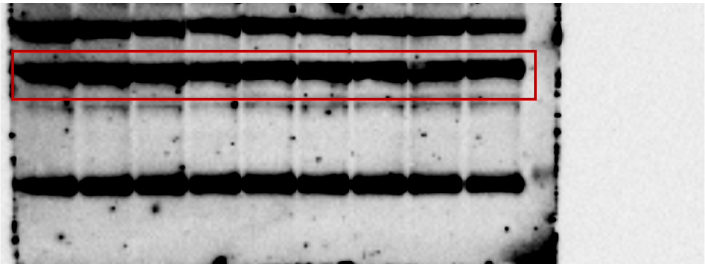

Blot 2 Lines 1-4 CTRL; Lines 5-9 HnB
a-tubulin

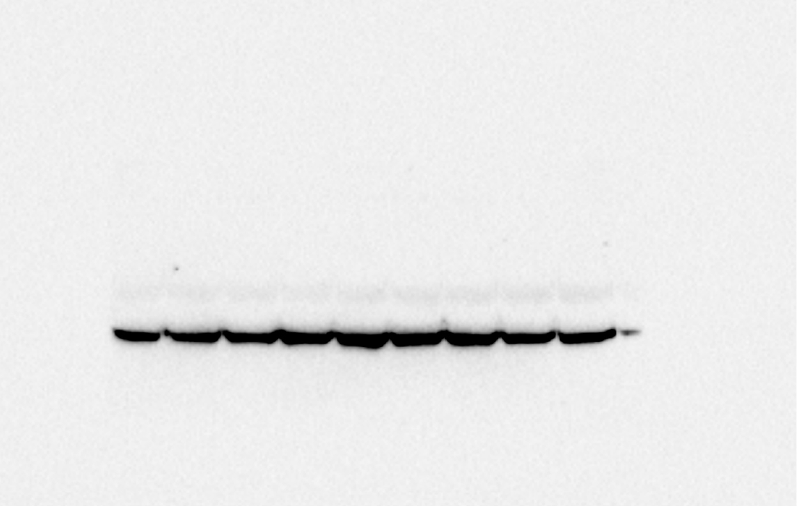

Blot 1 Lines 1-5 HnB; Line 4-9 CTRL
cMYC

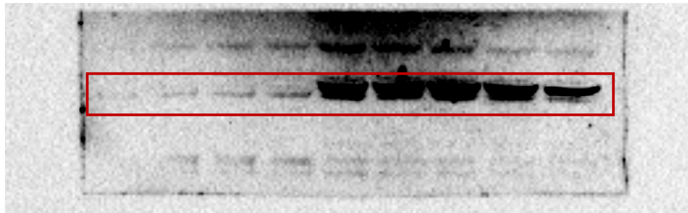

Blot 2 Lines 1-4 CTRL; Lines 5-9 HnB
COX-2

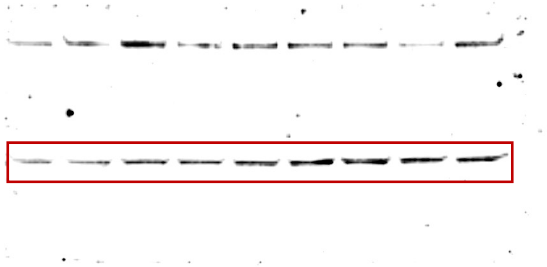

Blot 1 Lines 1-4 CTRL; Line 5-9 HnB
a-tubulin

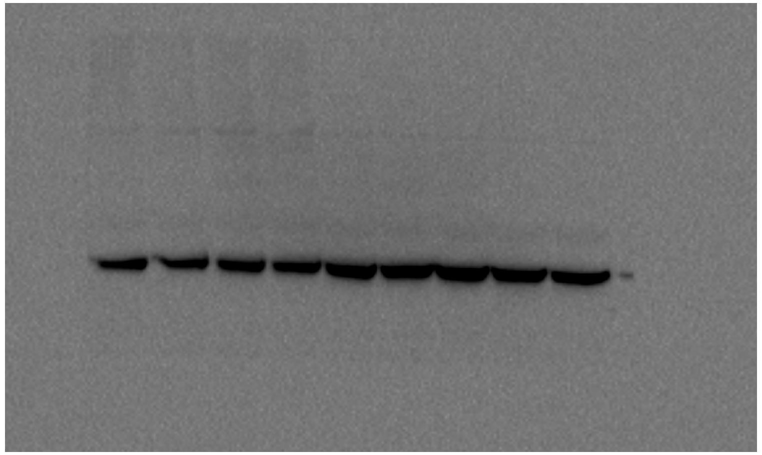

Blot 2 Lines 1-4 CTRL; Lines 5-9 HnB
a-tubulin

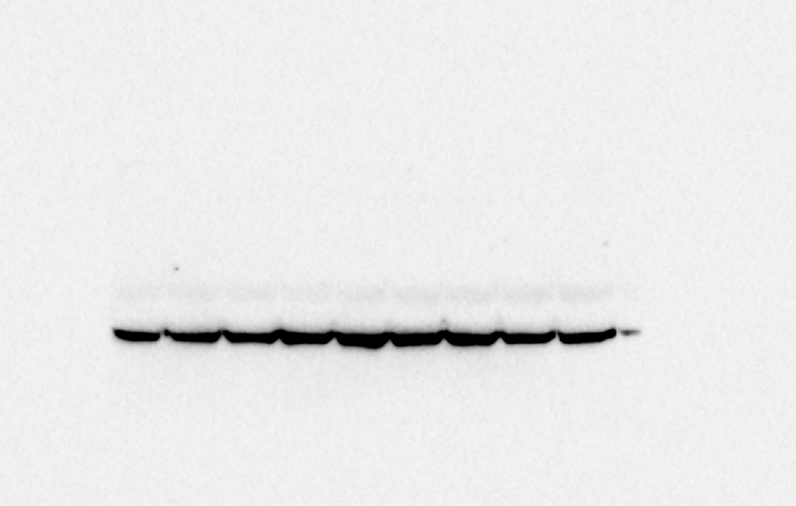

Blot 1 Lines 1-5 HnB; Line 6-9 CTRL
p-p38

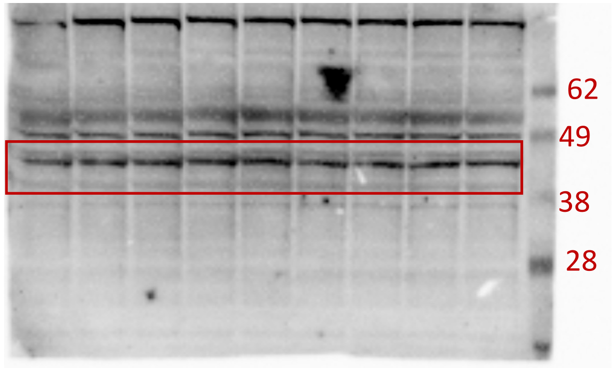

Blot 2 Lines 1-4 CTRL; Lines 5-6 HnB
p-38

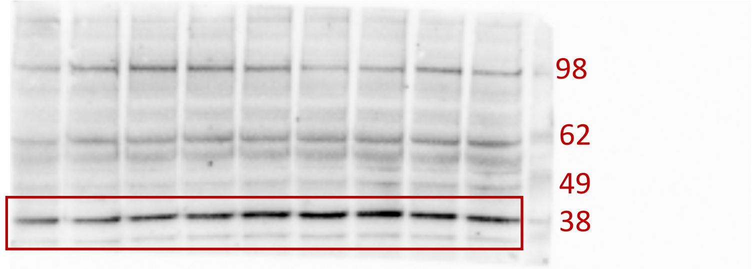

Blot 1 Lines 1-5 HnB; Line 6-9 CTRL
Tot-p38

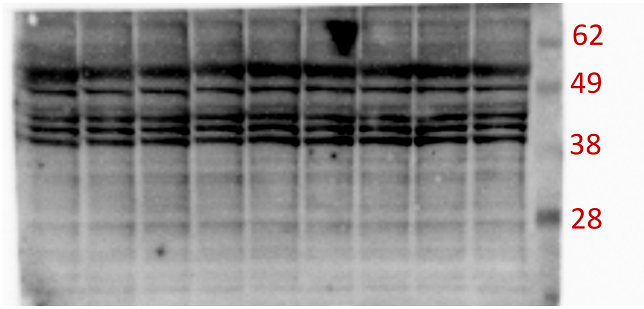

Blot 1 Lines 1-4 CTRL; Lines 5-6 HnB
Tot-p38

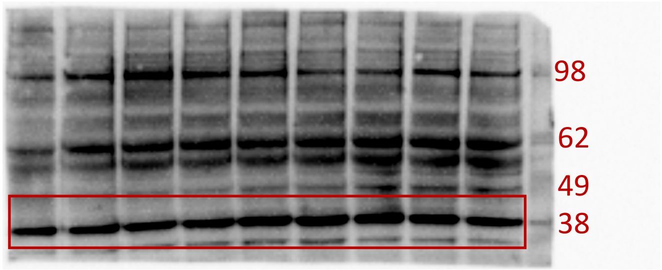

Blot 1 Lines 1-5 HnB; Line 6-9 CTRL
a-tubulin

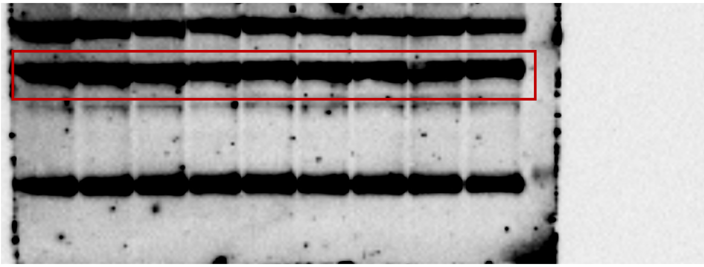

Blot 2 Lines 1-4 CTRL; Lines 5-6 HnB
a-tubulin

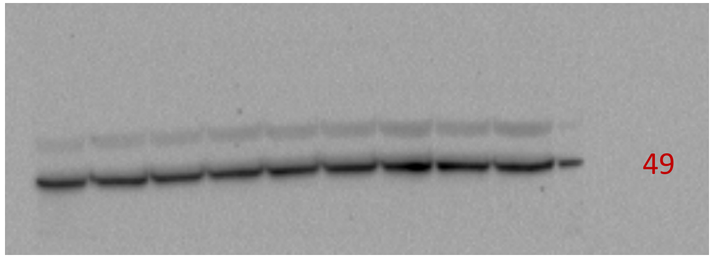

Supplement: Supplementary file 1 [file Image1.pdf]
